# The Role of Bottom-Up and Top-Down Cortical Interactions in Adaptation to Natural Scene Statistics

**DOI:** 10.3389/fncir.2019.00009

**Published:** 2019-02-13

**Authors:** Selam W. Habtegiorgis, Christian Jarvers, Katharina Rifai, Heiko Neumann, Siegfried Wahl

**Affiliations:** ^1^Institute for Ophthalmic Research, University of Tübingen, Tübingen, Germany; ^2^Faculty of Engineering, Computer Sciences and Psychology, Institute of Neural Information Processing, Ulm University, Ulm, Germany; ^3^Carl Zeiss Vision International GmbH, Aalen, Germany

**Keywords:** adaptation, visual system, distortions, dynamic synapses, motion, natural scenes, feedforward-feedback pathways

## Abstract

Adaptation is a mechanism by which cortical neurons adjust their responses according to recently viewed stimuli. Visual information is processed in a circuit formed by feedforward (FF) and feedback (FB) synaptic connections of neurons in different cortical layers. Here, the functional role of FF-FB streams and their synaptic dynamics in adaptation to natural stimuli is assessed in psychophysics and neural model. We propose a cortical model which predicts psychophysically observed motion adaptation aftereffects (MAE) after exposure to geometrically distorted natural image sequences. The model comprises direction selective neurons in V1 and MT connected by recurrent FF and FB dynamic synapses. Psychophysically plausible model MAEs were obtained from synaptic changes within neurons tuned to salient direction signals of the broadband natural input. It is conceived that, motion disambiguation by FF-FB interactions is critical to encode this salient information. Moreover, only FF-FB dynamic synapses operating at distinct rates predicted psychophysical MAEs at different adaptation time-scales which could not be accounted for by single rate dynamic synapses in either of the streams. Recurrent FF-FB pathways thereby play a role during adaptation in a natural environment, specifically in inducing multilevel cortical plasticity to salient information and in mediating adaptation at different time-scales.

## Introduction

Our visual perception is always affected by what we have observed in the past. For instance, after watching a moving entity after a prolonged amount of time, e.g., sea waves, stationary objects appear to move. Neurons in the visual system decrease their sensitivity after prolonged exposure to a specific type of visual input resulting in such concomitant perceptual modification for subsequently viewed stimulus (Blakemore and Campbell, 1969; Mather et al., 1998; Fang et al., 2005; Clifford et al., 2007). This experience dependent change in the visual system is called adaptation. Adaptation has a key role for optimal and stable perception in a continuously changing natural environment (Clifford et al., 2000; Kohn, 2007; Webster, 2011, 2015).

The natural environment is characterized by large variations of several attributes (Dong and Atick, 1995; Billock et al., 2001; Betsch et al., 2004; Bex et al., 2005, 2007, 2009). The design of the visual system is likely to mirror and efficiently compute the statistical properties of these attributes (Eckert and Zeil, 2001; Simoncelli, 2003; Kayser et al., 2004; Geisler, 2008; Snow et al., 2017). Visual information is processed in a cortical circuit formed by synaptic organization of feedforward (FF) and feedback (FB) connections between different cortical areas (Sillito et al., 2006; Mather et al., 2008; Stuit, 2009; Rokszin et al., 2010). One form of FF-FB recurrent interaction is a driving FF input from lower to higher cortical areas and a re-entrant modulatory FB enhancing the input signal matching to responses of higher cortical areas (Friston and Büchel, 2000; Hupe et al., 2001; Sillito et al., 2006). The FB modulation acts as a prediction signal from higher to lower cortical areas and allows propagation of disambiguated signals through the network. This benefits to extract global salient information from a noisy input during response normalization process among a pool of neurons (Friston and Büchel, 2000; Hupe et al., 2001; Sillito et al., 2006). Yet, the functional role of FF-FB recurrent interaction is not resolved for adaptation to features of the natural environment which are broadband and noisy.

Another unexplored functional relevance of reciprocal visual streams to adaptation is their synaptic dynamics. Activity dependent changes in synaptic inputs are one of the underlying mechanisms for adaptation (Abbott et al., 1997; Webster, 2015). The visual system adapts to changes in the environment that last for a wide range of durations. How synaptic adaptations in the FF and FB connections mediate adaptation at different time-scales is an open question. Specifically, if synaptic adaptation at a single rate in either of the streams or at distinct rates in both streams underlie adaptation is yet unknown.

In the present study, we investigated the functional relevance of FF-FB cortical organization to the adaptation processes during natural viewing with psychophysical experiments and model simulations. Statistics of different features in the natural environment is often altered by geometric distortions of daily used optical elements. Two prominent examples of distorting optical elements are progressive addition lenses (PALs) and VR displays. The distortions in these optical elements introduce perceptual discomforts in a significant amount of wearers (Barrett, 2004; Sheedy and Andre, 2005; Johnson et al., 2007; Meister and Fisher, 2008; Yao et al., 2014; Bashiri et al., 2017). However, after prolonged use, wearers report vanishing of the side effects indicating adaptation (Barrett, 2004; Yao et al., 2014; Alvarez et al., 2017). In this contribution, adaptation to distortion induced alterations is thereby used as a model system to address day to day visual experiences in a large number of populations who benefit from these optical elements (Keshner, 2004; Holden et al., 2008; Meister and Fisher, 2008; Laver et al., 2012; Aller, 2013; Bashiri et al., 2017).

Distortions prominently alter motion direction statistics of the natural visual input, e.g., skew geometric distortion in PALs (Meister and Fisher, 2008). Accurate motion perception has a key role in successful interaction with the dynamic natural world, be it in the inference of the direction of moving entities, or navigation through the environment. Its alteration by image skew is possibly one of the causes for the difficulties experienced by novice PAL wearers, like spatial disorientation during navigation (Johnson et al., 2007). Visual adaptation to distortion induced alteration in motion direction statistics of the natural visual world is thus essential to successfully use such optical utilities. In this representative example, we assessed the functional role of recurrent streams to adaptation within motion processing cortical areas, in particular to aspects of neural response tuning and time-scales of adaptation.

We quantified motion direction statistics of skewed and un-skewed natural image sequences to assess the skew effect. Adaptation to skew induced motion alteration was probed in psychophysical experiments by persisting perceptual adjustments, i.e., motion direction adaptation aftereffects (MAE), after exposure to the skewed natural stimuli at different time-scales. Our model architecture is based on a biologically inspired model of recurrent visual motion processing in the dorsal pathway of the visual system, namely in V1 and MT, comprising FF-FB pathways and activity normalization in each model area (Bayerl and Neumann, 2004, 2007; Raudies and Neumann, 2010a; Bouecke et al., 2011). We suggest dynamic synapses within direction processing intra-cortical circuitry as adaptive mechanisms. Assessing neural responses across V1 and MT model units with and without FB stream, physiologically plausible response tuning was observed during and after adaptation only when FB was integrated. FF-FB interaction additionally leads to synaptic adaptation within neurons selective to a salient motion direction signal by enhancing it over the noisy natural input. Furthermore, comparing prediction performance of different variants of the suggested model, the psychophysically observed MAE at different time-scales are best predicted by distinct adaptive mechanisms in FF and FB streams than a single adaptive mechanism in either FF or FF-FB circuitry. In sum, recurrent bottom-up and top-down cortical streams are integral parts of adaptation in a natural environment and multiple dynamic synapses operating at different time scales within the reciprocal streams mediate temporal context dependency of this adaptation.

## Results

### Image Skew Alters Motion Direction Statistics of Natural Image Sequences

**Figure 1:**
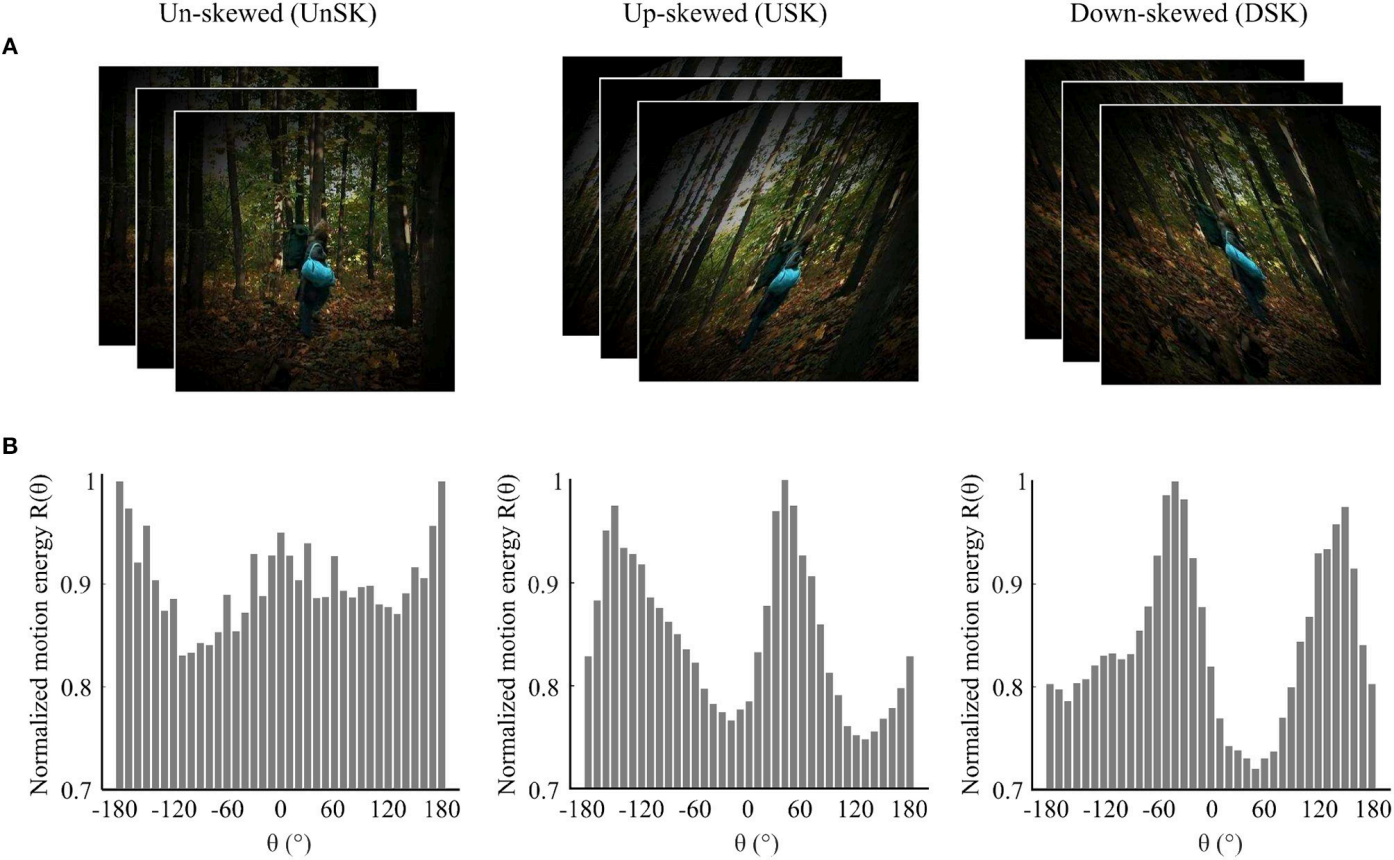
Illustration of the effect of image skew on motion direction statistics of natural image sequences. **(A)** Examples of un-skewed (UnSK), up-skewed (USK) and down-skewed (DSK) natural images that are weighted by a circular shape Hanning window. **(B)** Average motion energy from 12,000 natural image sequences as a function of motion direction (θ). Motion direction statistics of UnSK, USK and DSK natural image sequences was detected by correlation based Elaborated Reichardt detectors (ERDs) (Reichardt, 1987; Bayerl and Neumann, 2004). Negative θ represents down-ward and positive θ represents up-ward motion directions.

Natural image sequences were acquired from an open source movie as they are exemplary for everyday visual input. Opposite skew geometric distortions, i.e., up-skew (USK) and down-skew (DSK), were simulated in the images. The average motion direction statistics of the skewed and un-skewed image sequences were quantified by correlation based Reichardt motion detectors. Detailed descriptions of distortion simulation and motion detection are provided in the Methods section and Supplementary Materials, respectively. As illustrated in Figure 1, motion direction signal statistics in the un-skewed image sequence is broad band but dominated by horizontal direction signals. In the skewed natural image sequences, the dominant signal is shifted in the skewing direction, i.e., to the positive in USK stimuli and to the negative in the DSK stimuli.

### Evaluation of Skew Induced MAE

MAE was tested after sequential exposure to DSK then USK natural image sequences. Adaptation aftereffects of each adapting skew direction were tested by the motion direction of a coherently moving random dot test stimuli that was perceived as horizontal, i.e., equally likely to be upward and downward in a method of constant stimulus procedure. From here on, we refer to this parameter as point of subjective equality (PSE).

**Figure 2:**
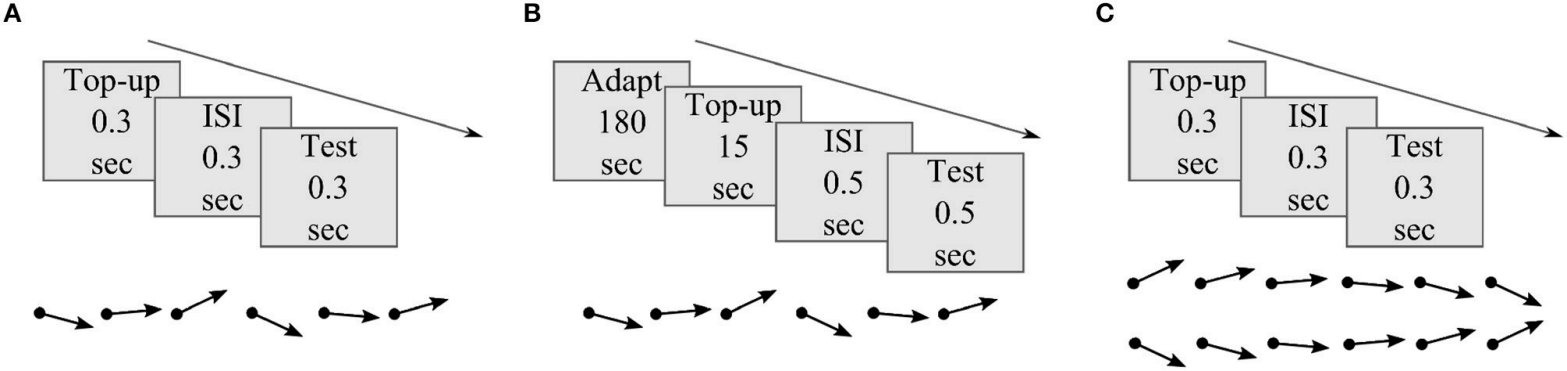
Basic illustration of MAE measurement experiments. **(A)** Experiment 1: MAE after short skew exposure; each adapting skew top-up and test stimuli presentation lasted for 0.3 s separated by same duration of black screen presentation in the inter stimulus interval (ISI) period. b) Experiment 2: MAE after long skew exposure; prior 180 s of skew adaptation followed by 15 s of top-up, 0.5 s of ISI and 0.5 s of test stimuli presentation. c) Experiment 3: MAE after short skew exposure and hysteresis effect; each top-up,ISI and test stimuli presentation lasted for 0.3 s. The dot ended arrows represent if the order of the test stimuli between successive trials was randomized, in **(A)** and **(B)**, or sequential, in **(C)**.

Figure 2 illustrates the basic schematics of the three psychophysical experiments used to evaluate distortion induced adaptation. Experiment 1 and 2 were designed to test MAE after short and long timescales of skew exposure with randomized order of test stimuli motion direction. The results of these experiments were used to fit model parameters and to test prediction performances at different timescales. In experiment 3, MAE was tested after short skew exposure with sequentially increasing or decreasing order of test stimuli motion direction to introduce additional hysteresis effect, i.e., from down to up direction for the DSK adaptation and from up to down for the USK adaptation. Perceptual hysteresis occurs when motion direction of an input stimulus changes gradually, e.g., from up to down direction or vice versa, due to internal short term memory of the underling neural network as was previously demonstrated in computational findings (Williams and Phillips, 1987; Bayerl and Neumann, 2004). Accordingly, the results of experiment 3 were used as an additional dataset to validate the models' predictions and in particular contribution of FB in the network short term dynamics. Detailed explanation of the psychophysical experiment procedures are included in the Methods section.

### Distortion Induced MAE: Psychophysics

**Figure 3:**
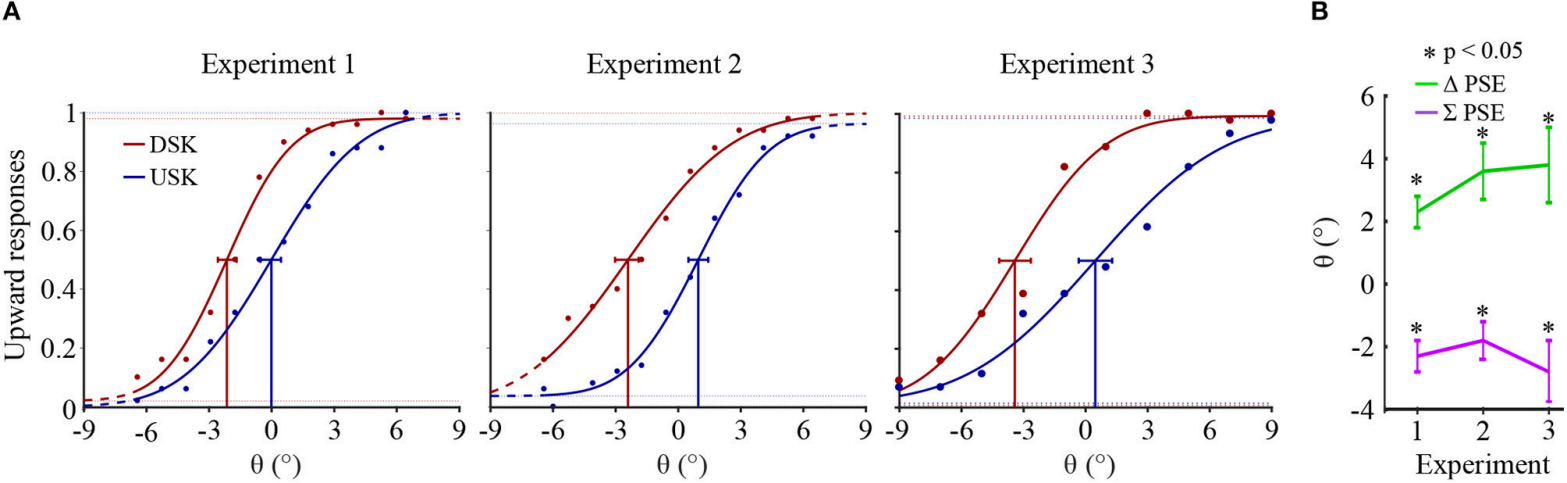
**(A)** Psychometric functions of overall observers' responses in the three experiments. In each psychometric plot, the Gaussian fitted plot and the confidence intervals at PSE are, respectively shown in red for the DSK adaptation aftereffects and in blue for the USK adaptation aftereffects. **(B)** Overall observers' averages of Δ*PSE* and ∑*PSE*. The error bars show the standard errors of the averages.

Figure 3 shows the psychometric curves of overall observers responses recorded after skew exposure in the three psychophysical experiments. The percentage of upward responses is plotted as a function of test stimulus motion direction, θ. A negative θ value corresponds to a downward motion direction and a positive value to an upward one.

The shift in the PSEs of the opposite skew adaptation, i.e., Δ*PSE* = *PSE*_*USK*_ − *PSE*_*DSK*_, was significantly positive in all the three experiments (paired sample *t*-test, *p* < 0.05). After adaptation to up-skewed natural stimuli, observers perceived an upward motion direction as horizontal and vice versa. Thus, the PSE shifted to the direction of the adapting skew whereas the physical horizontal motion direction is perceived to be shifted in the opposite direction. Furthermore, in all the experiments, the PSEs from the two oppositely skewed stimuli were not symmetric about the physical 0° of motion direction albeit the amount of the skew in the adapting stimuli was equal in magnitude. This is illustrated by a significant bias in the sum of the PSEs, ∑*PSE* = *PSE*_*USK*_ + *PSE*_*DSK*_, in all experiments toward the negative; paired sample *t*-test *p* < 0.05. The asymmetry conceivably reveals either the adaptation state induced by the first DSK exposure which was not recovered during the subsequent USK exposure or due to a relative difference in the strength of the DSK than the USK adapting signal, where both are not mutually exclusive.

### Model: Dynamic Synapses Within a Recurrent Motion Processing Cortical Circuit

Activity dependent short term synaptic depression is one potential source of neural response adjustments during adaptation (Castellucci et al., 1970; Abbott et al., 1997; Kohn, 2007; Regehr, 2012; Tetzlaff et al., 2012; Webster, 2015). The strength of synaptic coupling between cells, i.e., synaptic efficacy, depends on the presynaptic vesicle occupancy which is the amount of releasable neurotransmitters (Hennig, 2013). Repeated activation of the post synaptic cell by the presynaptic cell causes depletion of these vesicles and a concomitant reduction in postsynaptic response (Hawkins et al., 1993; Zucker and Regehr, 2002; Hennig, 2013). If the excitation input is removed from the presynaptic neuron, the synaptic strength will slowly restore until the vesicles are fully replenished. Short term synaptic depression has been previously revealed in different brain areas and its potential implications in various dynamics of recurrent cortical neural networks have been reported (Abbott et al., 1997; Tsodyks et al., 1998; van Rossum et al., 2008; York and Van Rossum, 2009; MacLeod et al., 2010; Stevenson et al., 2010; Jääskeläinen et al., 2011). The ubiquity of short term synaptic depression in several of cortical areas, reflects that it is a characteristic mechanism across cortical circuitries underlying neural response adjustments during adaptation. Such dynamic synaptic mechanism in motion processing cortical circuitry potentially underlies the psychophysically demonstrated distortion induced MAE. Wherein, during adaptation, exposure to distorted motion direction information decreases the synaptic strength between neurons selective to those directions. Subsequently, when test stimuli are presented before the synaptic strength is replenished, an aftereffect can be observed. Accordingly, such synapse specific gain control mechanism within a recurrent motion processing cortical circuitry is considered in our model to investigate response adjustments of motion processing neurons during exposure to motion information altered by distortions.

Our model extends a previously developed architecture for recurrent motion processing in V1 and MT areas comprising FF and FB recurrent streams (Bayerl and Neumann, 2004). This model framework has been selected as it considers a characteristic feature of the cortical architecture, i.e., bidirectionally connected cortical areas, which would allow us to assess the relative contribution of the reciprocal streams for the adaptation process. Furthermore, this model has been shown to be able to predict data from neurophysiological recordings as well as behavioral studies (Bayerl and Neumann, 2004, 2007; Raudies and Neumann, 2010a; Bouecke et al., 2011). Direction tuned neural activation is described by a membrane potential of a single compartment column model that depends on excitatory, modulatory and inhibitory synaptic inputs. As an extension, dynamic synaptic efficacies were considered in the FF and FB excitatory connections to introduce synaptic adaptation in the model.

**Figure 4:**
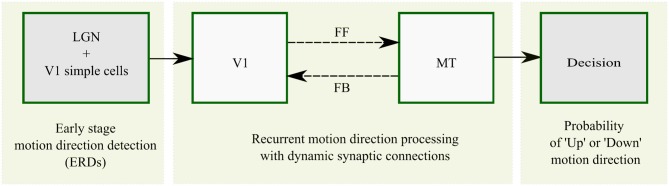
Overview of recurrent motion processing in cascaded model areas and their interactions. Motion is initially detected from image sequences in sequential early stage preprocessing by LGN and V1 simple cells which is realized by ERDs. Motion direction signals are subsequently processed in a recurrent loop of direction selective cells in V1 and MT connected by FF and FB streams. This stage is the core component of the model where synaptic plasticity is introduced within the FF and FB streams as highlighted by the broken lines. A steady-state model of decision-making finally maps MT responses into “upward” and “downward” responses.

A scheme for the basic architecture of the model in cascaded motion processing at different cortical areas is illustrated in Figure 4. The model starts with preprocessing of the input by LGN cells followed by motion direction signal detection by direction selective filters in V1 that are implemented as spatiotemporal correlation techniques (ERDs). The subsequent recurrent motion direction processing by direction selective cells within areas V1 and MT is the main component of the model where synaptic plasticity is implemented. In this stage, dynamic synapses within reciprocal FF and FB streams are taken into account to introduce plasticity. The dynamic synapse constitutes short-term synaptic adaptation as a consequence of vesicle depletion and replenishment that occur during the presence and absence of excitatory presynaptic signals, respectively. This is realized by dynamic synaptic efficacy parameters that weight the FF and FB excitatory inputs from V1 to MT and from MT to V1, respectively. As a final stage, a steady-state model of decision-making layer maps MT responses into “upward” and “downward” responses in order to account for the decision process employed in the psychophysical experiments. The computational modeling of the synaptic efficacies and neural activations is described in detail in the Methods section.

**Figure 5:**
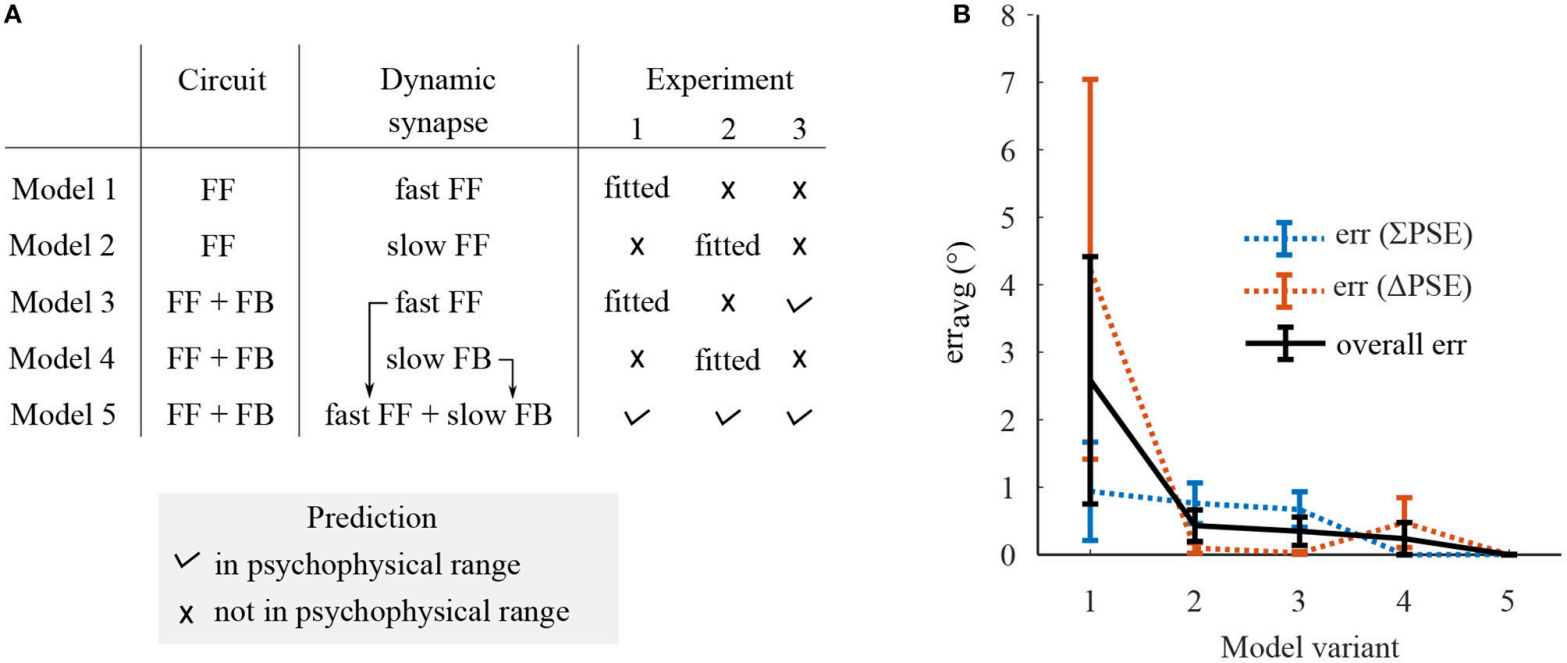
A summary on prediction performance of five model variants comprising dynamic synapses operating at either single or multiple rate in either only FF or FF-FB cortical circuitries. **(A)** The experiments in which the model variants predict the Δ*PSEs* and ∑*PSEs* within the range of psychophysical results or not. **(B)** The prediction error, *err*_*avg*_, which was defined as the averaged magnitude of the deviations of each model's predictions from the corresponding psychophysical ranges.

Previous findings reported that adaptation at different timescales is mediated by multiple adaptive mechanisms which operate at distinct rates (Mesik et al., 2013). Specifically, these findings suggested that a fast adaptive mechanism, which adapts and de-adapts fast, and a slow adaptive mechanism, which adapts and de-adapts slow, coexist in a cortical circuit to mediate adaptation at different timescales. In line with these findings, in our model variants, distinct fast and slow dynamic synapses tuned to different adaptation rates are considered. As explained in detail in the following paragraph, in the fast dynamic synapses, the rates of vesicle depletion and replenishment were fitted to replicate MAE from milliseconds of skew exposure in experiment 1. In the slow dynamic synapses, the rates of vesicle depletion and replenishment were fitted to replicate MAE time scales of minutes of skew exposure in experiment 2 (see Methods section). To test if the synaptic dynamics in the recurrent FF-FB streams could underlie these findings, predictions of MAEs in the three psychophysical experiments were tested from five different model variants. As illustrated in Figure 5A, the model variants were defined by considering different complexity of the circuitry, either only FF or FF-FB, and different temporal rate dynamic synapses, either single or multiple dynamic synapses. Adaptation is commonly modeled as a reduction in input gain or changes in the strength of normalization within a single cortical area without recurrent connectivity. Using this approach as a baseline, model variant 1 and model variant 2 comprise a simple FF circuit with a dynamic synapse which changes the response gains of MT responses. The rates of the dynamic synapses in model variant 1 and model variant 2 were fast and slow which were fitted to replicate the psychophysical results of experiment 1 and experiment 2, respectively. To compare the recurrent FB effect, model variant 3 and 4 were defined by considering a FB circuit. Analogous to the first two model variants, model variant 3 and model variant 4 comprise either fast FF and slow FB dynamic synapses fitted to replicate psychophysical results of experiment 1 and experiment 2, respectively. With the presumption that FF mechanisms are faster than feedback mechanisms (Destexhe et al., 1998; Roth and van Rossum, 2009; Thiele, 2012), fast dynamic synapses are considered in the FF stream and slow dynamic synapses are considered in the FB stream. In contrast to the above four model variants comprising single adaptive mechanism, model variant 5 was defined by considering dynamic synapses in both FF and FB circuits which operates at distinct timescales. The rates of its FF and FB dynamic synapses were taken from model variant 3 and model variant 4, respectively. The prediction performance of each model variant was validated with either experiment 1 or experiment 2 to which they were not fitted to and with an additional data set from experiment 3. Finally, the role of FF-FB interaction on neural response tuning properties has been investigated by comparing neural responses of model variant 1 and model variant 5 in the absence and presence of FB, respectively.

### FF-FB Functional Role in Adaptation at Multiple Time-Scales

The human visual system optimally adapts to the changes in the natural environment which span different timescales. As suggested by previous studies, multiple adaptive mechanisms potentially underlie adaptation at different timescales (Bao and Engel, 2012; Bao et al., 2013; Mei et al., 2015). However, if these multiple adaptive mechanisms correspond to the dynamic synapses in the recurrent FF-FB streams of the cortical circuitry has not been previously assessed. To test the functional relevance of FF-FB circuits, specifically their synaptic dynamics for mediating MAE at different time-scales, we compared prediction performance of the first four model variants with dynamic synapses operating at a single rate to model variant 5 which entails different rate dynamic synapses in the FF and FB circuits.

A summarized prediction performance of each model variant is shown in Figure 5. The psychometric curves of model predictions together with the psychophysical results are presented in the Supplementary Materials. Model variants constituting a single adaptive mechanism, variant 1–4, does not predict Δ*PSEs* and ∑*PSEs* at the time scales of adaptation other than they are fitted to. Model variant 1, comprising FF circuit with the fast dynamic synapse, does not predict any of the experiments and gives the largest prediction error. The fast synaptic mechanism of model variant 3 predicts only short adaptation MAEs in experiment 1 and 3 and not the MAEs from the long exposure in experiment 2. The slow dynamic synapses of model variants 3 and 4 predict the MAEs only from long exposure in experiment 2 but not from the short exposures in experiment 1 and 2. Model variant 5, comprising distinct fast and slow dynamic synapses in FF and FB streams, predicts all the three skew induced MAEs at the different time-scales. From the overall prediction, this model variant performed best. Furthermore, among the model variants with fast dynamic synapses, only those with FB circuit predicted experiment 3 albeit all were fitted to that specific adaptation timescales. This indicates FB is an integral part of the neural network underlying adaptation.

In sum, a FF-FB model with slow and fast adaptive mechanisms best predicts skew induced MAE at different time-scales which cannot fully be accounted by a single adaptive mechanisms in only FF or FF-FB circuit. Thus, the synaptic dynamics of FF-FB cortical circuitries tuned at different time-scales are relevant in mediating adaptation at different time scales during natural vision.

### FF-FB Functional Role in Neural Response Tuning

The input from the natural environment consists of uncertainties or noise as is depicted in Figure 1. The visual system however adapts in an optimal manner by inferring relevant information from such uncertainties using prior knowledge of the environment (Kohn, 2007; Wark et al., 2009; Stevenson et al., 2010). Since FB acts as such predictive signal from higher to lower cortical areas, it possibly plays a role in such optimal adaptation, but this has not been previously demonstrated. Here, to test the role of FF-FB interaction in neural response tuning during adaptation, we assessed responses of V1 and MT model units during and after adaptation in model variant 1 and 5, i.e., in the absence and presence of FB circuitry, respectively.

**Figure 6:**
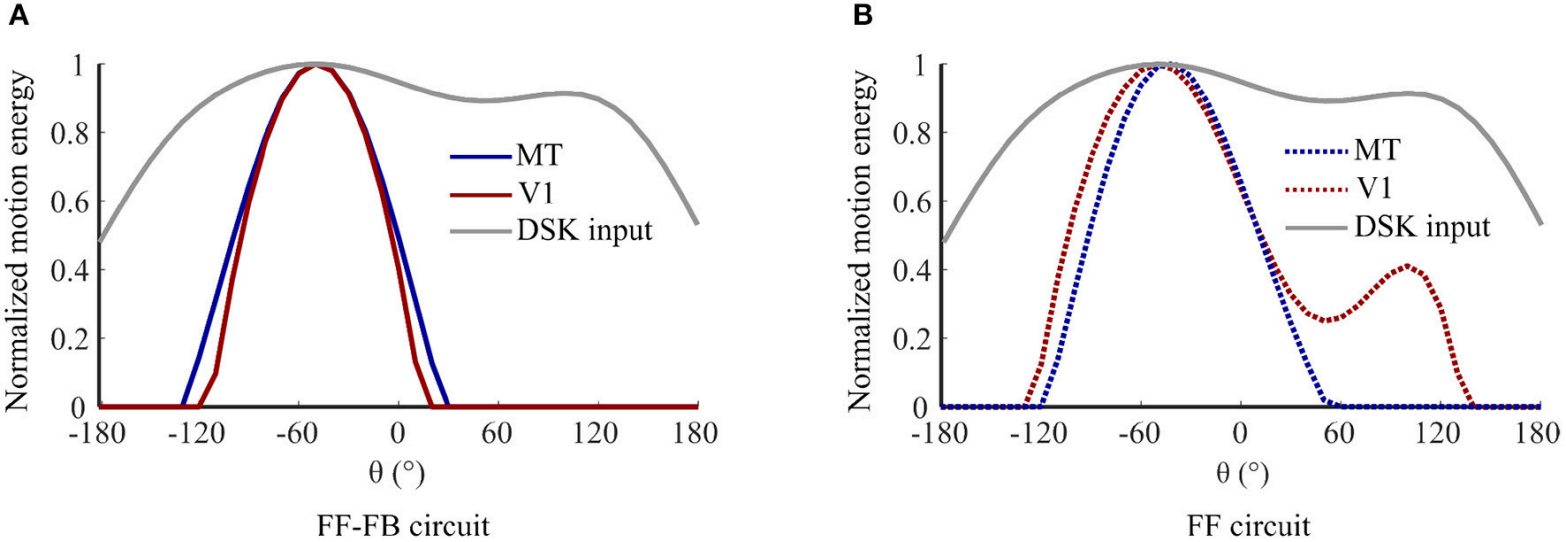
Contribution of FB in enhancing salient motion information: normalized response curves of V1 and MT model units after 0.3 s exposure to DSK adapting stimuli a) in model variant 5, i.e., FF-FB circuit and **(B)** in model variant 1, i.e., only FF circuit.

Figure 6 shows an example simulation of model units' responses in V1 and MT during adaptation to DSK stimuli for 0.3 s. In model variant 5, V1 and MT responses are similar and have a uni-modal response patterns with peaks at the salient information of the input. In model variant 1, i.e., when FB is eliminated, the response curves of V1 and MT model units have a different pattern and maxima. V1 response is bimodal and resembles the pattern of the noisy input statistics. Whereas, the MT response is uni modal with a maximum between the two modes of V1 response pattern. Thus, FB disambiguates salient motion information from the noisy input and results in similar response tuning in model area V1 and MT during adaptation.

**Figure 7:**
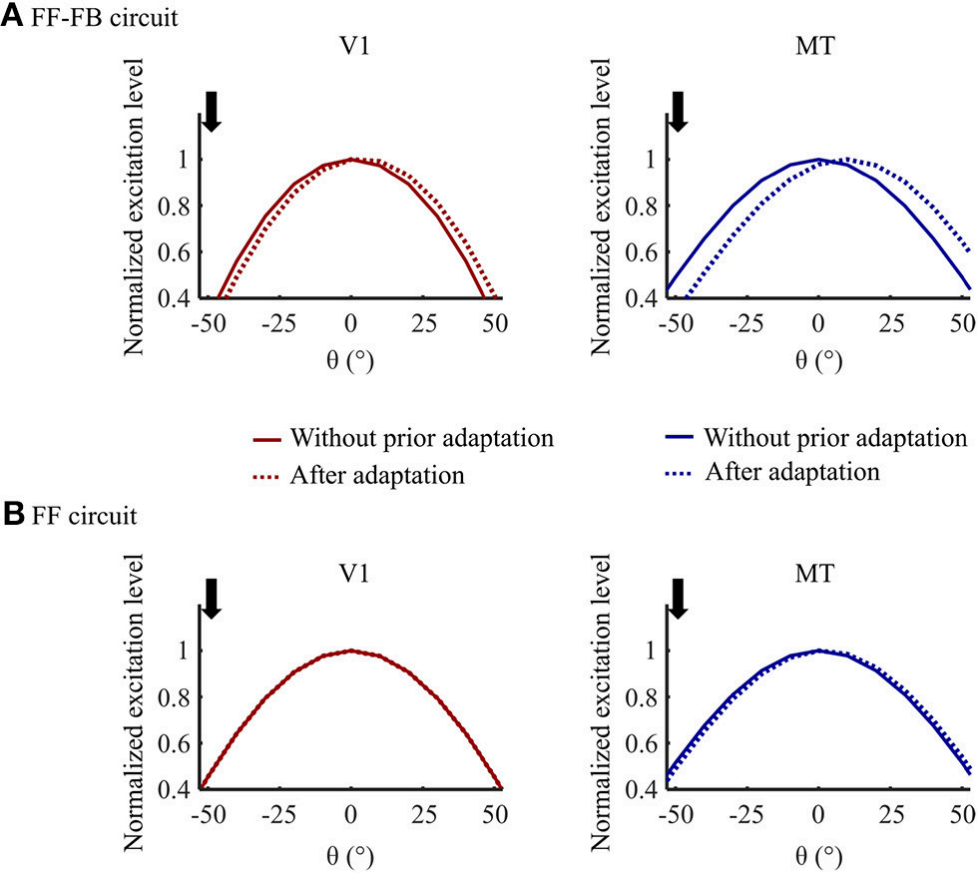
Adaptation effect comparison in **(A)** Model variant 5 (FF-FB) and **(B)** Model variant 1 (only FF). Normalized response curves of V1 and MT model units for the test stimuli input at 0° motion direction without prior adaptation and after 0.3 s of DSK adaptation. The downward arrow indicates the dominant motion direction, at –50°, in the adapting DSK stimuli.

An example simulation of V1 and MT model units' responses for 0° test stimulus motion direction without and with prior DSK adaptation are illustrated in Figure 7. In model variant 5, exposure to DSK adapting stimuli shifted the response curves of both V1 and MT units toward the up direction away from their preferred direction which was measured without prior adaptation. The FB dynamic synapse is slow, thus not depleted enough by such short exposure time-scale to induce adaptation effect in V1. The fast FF dynamic synapse tuned at short time-scale induces adaptation to the MT responses. The recurrent FB projection which modulates the V1 input matching to the adapted MT response induces the repulsive effect in V1. In model variant 1, however, the repulsive aftereffect is visible only in MT model units since the dynamic synapse in the FF circuit modulates only the input to MT. Furthermore, the repulsive effect in this variant is smaller than what is observed in model variant 5. This indicates compensatory adaptation effect of the V1 bimodal response pattern during DSK exposure in model variant 1. Thus, the inclusion of FB connections results in similar adaptation to a salient motion information in model area V1 and MT.

In sum, exposure to skewed natural image sequences results in repulsion of response curves in model areas V1 and MT. This neural response tuning conceivably underlies skew adaptation.

## Discussion

Skew induced MAE and the underlying cortical processes were assessed with psychophysical experiments and model simulations. MAE was revealed at two time scales of skew exposures in both psychophysical results and model predictions. The observers PSE, i.e., random dots motion direction perceived as horizontal, shifted in the adapting skew direction. In other words, the physical horizontal motion direction of the test stimuli was perceived to be shifted away from the adapting skew direction. Thus, exposure to skewed natural stimuli induces adaptation in motion perception which is a candidate mechanism for habituation to distortions of optical elements.

A recurrent neural model of two stage motion direction processing with distinct dynamic synaptic mechanisms within FF and FB connections was suggested to explain the potential underlying mechanism of the observed MAE. Simulation results of the model replicated the psychophysically shown MAE at different time scales of skew exposure. Thus, multiple rate adaptive mechanisms within recurrent FF and FB cortical pathways explain adaptation to optical manipulations of the natural environment, e.g., through distortion by optical elements.

### Biological Relevance of the Suggested Model

Unlike pure feedforward mechanisms considered in previous adaptation models(Clifford and Langley, 1996; van de Grind et al., 2003), motion information is processed in a cortical circuit formed by synaptic organization of FF and FB connections between different cortical areas (Sillito et al., 2006; Mather et al., 2008; Stuit, 2009; Rokszin et al., 2010). A modulatory feedback re-entrant mechanism suggested in the present study has been reported in previous physiological investigations (Friston and Büchel, 2000; Hupe et al., 2001; Sillito et al., 2006). Specifically, excitatory FB projection from MT combined with activity normalization by subsequent center surround competition was shown to affect activities of V1 cells.

One important aspect of top-down FB signal is the disambiguation of motion information via selective enhancement and propagation of salient signals (Bayerl and Neumann, 2004, 2007; Raudies and Neumann, 2010a; Bouecke et al., 2011). The motion direction signal from the adapting natural image sequences are highly ambiguous and noisy as shown in Figure 1. In our model, these ambiguous signals are reduced and salient direction information is enhanced as a result of feedback and lateral competitive interactions. Figure 6 shows this disambiguation in activities of V1 and MT in the presence and absence of such FB mechanism. The FB functions as a predictive signal that enhances lower level input that matches “expectations” or feature specificity of higher level responses (Grossberg, 1982; Ullman, 1995). In this manner, only the input signal that is in resonance to the higher level MT response is enhanced. As a consequence, in line with physiological findings, FB disambiguates motion direction signal simultaneously in both V1 and MT areas (Pack et al., 2003). Moreover, when FB connections are integrated into the model, similar adaptation induced response tuning occurs in both V1 and MT areas, consistent with physiological findings (Patterson et al., 2013), see Figure 6. If the FB pathway is eliminated, a repulsive shift adaptation effect would be visible only in MT model units since the FF dynamic synapses affect only the driving FF input stream from V1 to MT. Thus, synaptic adaptation within FF-FB connections reflects physiological plasticity in different visual areas during skew adaptation.

The model simulation results also align with Bayesian inference prediction frameworks of optimal adaptation processes which necessitate predictive prior information (Grzywacz and de Juan, 2003; Kording et al., 2007; Wark et al., 2007, 2009). A comparable resemblance with such model can be derived by considering the receptive fields and dynamic synaptic strengths as adaptive likelihood functions and the feedback information as an adaptive prior information. However, in the present model, priors are dynamic over time and the likelihoods are not statistically independent. As FB allows predictive prior information from MT to modulate the likelihood of V1 responses, disambiguated motion signal will propagate though the network until a global consistent information in both areas is achieved. In line with the inference theory, this leads to optimal adaptation to a salient information by decreasing the risk of adapting to error signals. In this case, the suggested neural model can be treated as a neuronal implementation of Bayesian-like principles of adaptation.

Furthermore, the FB re-entrant mechanism is one way of integrating multiple adaptive mechanisms which control adaptation at different time-scales. Previous models assume motion adaptation to be a consequence of a single adaptive mechanism that operates at a specific time scale within a hierarchical bottom-up motion processing cortical circuit (Clifford and Langley, 1996; van de Grind et al., 2003). However, recent psychophysical findings reported evidence on distinct multiple adaptation mechanisms operating at different time scales (Mesik et al., 2013). In our model, we incorporated two different temporally tuned adaptive mechanisms, FF synapses tuned to short adaptation and the FB synapses to long adaptation. This enabled accurate prediction of skew induced MAE at different time-scales. To examine if skew induced MAE could be explained by one of the single adaptive mechanisms, we compared prediction performance of our model with its variants which comprise only a single adaptive mechanism. The simulation comparison shows that the prediction performance critically depends on FF-FB recurrency with different adaptive mechanisms. Thus, multiple adaptive synapses within FF and FB streams of motion processing circuitry are candidate mechanisms for skew induced MAE operating at different time-scales.

The model employed in this investigation is detailed at a mesoscopic level of description regarding its structure and the computational elements. In particular, the state variable is defined by the mean activation (membrane potential) over a population of neurons equivalent to a cortical mini-column. Each mini-column represents a single feature selectivity at a specific spatial position. Different feature selectivities (motion direction, in our case) at one location comprise a model cortical column. The mean potential changes in such population are formally described by a first-order rate equation (Equation 4). The membrane time constant for individual cells in cortex varies for different types of neurons (Markram et al., 2004; Monier et al., 2008). Since we here consider the dynamics of whole populations of neurons in mini-columns we have adjusted the time constants to a value higher than that of individual neurons. This accounts for the variation response times in the population and the exerted effects of inhibition on the excitatory units.

The components considered in the model are minimal in the sense that structural elements which do not contribute to the considered functionality are not included. Specifically, the model investigation emphasizes short-term synaptic adaptation, which is adopted here by a habituative mechanism (Castellucci et al., 1970), and feedback from activity representations at a higher stage (MT) to an earlier stage (V1). In contrast, lateral connections between columns in a spatial neighborhood are not incorporated in the model, even though they are observed in anatomy. We argue that the adaptation of neuronal selectivity and its modulation by contextual feedback from higher stages are sufficient to explain motion adaptation in the experimental setting investigated here. Such feedback spreads over a larger spatial neighborhood than lateral intra-cortical connections and also acts on a shorter timescale which is comparable with the feedforward delivery of signals. Since lateral connections seem not to make a major contribution to the contextual skew adaptation effects investigated here, we did not incorporate lateral connections in our model.

Overall, we believe that the proposed model and its selected granularity capture the most relevant details of neural response characteristics at the primary stages of cortical motion adaptation. The characteristics of the model dynamics have been investigated from a theoretical perspective to characterize steady-state solutions and other temporal response properties (Brosch and Neumann, 2014). These insights helped us to specify a stable configuration of a reference model network and to integrate the model extensions. In a nutshell, the short-term adaptation of synaptic connections explains the desensitization of units (groups of neurons in a mini-column) and the shift of direction tuning at the population level. This allowed us to establish a link to the mesoscopic neuronal model dynamics to the behavioral response characteristics of human subjects adapted to skewed input patterns as measured psychophysically.

### Other Potential Neural Correlates for Distortion Induced MAE

Substantial information has been revealed in classical adaptation studies by testing adaptation effects induced by artificial stimuli such as random dots and bars, albeit this might not always divulge visual performances observed in natural viewing conditions (Ringach et al., 2002; David et al., 2004; Felsen and Dan, 2005). Natural image content is thereby an ideal stimulus to potently drive the visual system in its intended mode (Webster, 2015; Snow et al., 2017). To enhance the ecological relevance of the investigation, the present study tests distortion induced motion adaptation when the visual system is exposed to stimuli that mimic the dynamics of the natural environment.

Adaptation aftereffects originate from response changes in neurons processing common features of adapting and test stimuli (Clifford et al., 2007; Webster, 2011, 2015). Skew distortion of natural image sequences alters multiple features, e.g., motion direction statistics, orientation statistics and oblique magnification. Adaptation to such attribute rich stimuli might activate several cortical areas and could involve their coordinated responses (Habtegiorgis et al., 2017). The present study assessed MAE induced by distortions of natural scenes and predicted response changes in direction selective neurons across cortical areas V1 and MT. Motion is dominantly processed in the dorsal visual pathway, albeit inputs from form processing areas might as well contribute to motion information processing, and thus to skew-induced MAE (Geisler, 1999; Edwards and Crane, 2007; Beck and Neumann, 2010; Pavan et al., 2013). Our models scope covers the role of FF-FB streams and their synaptic dynamics to adaptation within motion direction selective neurons in the dorsal visual pathway. However, FF and FB reciprocal streams considered in the present study are characteristic means of information flow between different cortical layers (Felleman and Van, 1991; Bastos et al., 2015). Thus, the contributions revealed here might reflect a general adaptation mechanisms in other feature selective cortical areas as well.

## Conclusion

The present study investigated skew adaptation mechanisms for image sequences from a psychophysical as well as a modeling perspective. Skew distortions, as simulated here with natural image sequences, typically occur for viewers wearing spectacles or experiencing distortions after changes in eyesight. The results obtained from the psychophysical investigations illustrate that skew exposure in milliseconds and minutes time-scales induces adaptation in motion perception. In the computational modeling investigation, a recurrent FF-FB model of motion detection and integration was employed and further elaborated to include synaptic adaptation mechanisms in the bottom-up and top-down connections. The model responses replicated psychophysical findings of adaptation effects after different time-scales of skew exposure. In the model, the FF-FB interactions and their synaptic dynamics had distinct roles in the computation wherein together they lead to such psychophysically plausible adaptation responses. Furthermore, the models prediction on an additional hysteresis effect yielded similar results when tested psychophysically. This indicates analogous temporal dynamics of the neural networks in the suggested model and the human visual system underlying skew adaptation at multiple time-scales.

## Methods

### Psychophysics

#### Study Approval

The study was approved by the Ethics Committee of the Medical Faculty of the Eberhard Karls University of Tübingen and the University Hospital.

#### Observers

10 observers participated in experiment 1 and 2, among which six partook in both. 11 observers participated in experiment 3, eight of which also participated in experiment 1 and five of which in experiment 2. All observers aged between 18 to 40 years. All observers had normal or corrected to normal vision during the experiment and were naive about the purpose of the study. In adherence to the Declaration of Helsinki, informed written consent was collected from all observers before their participation in the study.

#### Set-Up

The psychophysical experiments were designed using PsychToolbox in Matlab (Mathworks, MA, USA) (Brainard, 1997). Stimuli were displayed on a ViewPixx/3D monitor at a resolution of 1920 × 1080 pixels (with 0.271 mm pixel pitch) and refresh rate of 100 Hz in an otherwise darkened room. A chin and head rest was used to fix the viewing distance to 57 cm. Up and down keys of a keyboard were used to collect observer's responses during adaptation aftereffect measurements.

#### Stimuli

12,000 natural images, of size 1, 280 × 720 pixels, were sequentially taken from an open source movie to prepare the skewed adapting stimuli (Baumann and Behnisch, 2010). Each natural image was geometrically skewed at a shear angle of Ψ in a horizontal and vertical directions by remapping pixel positions of undistorted image, *x* and *y*, into new distorted pixel positions, *x*_*d*_ and *y*_*d*_, as in Equation (1).

(1) $$\begin{array}{l} \left[\begin{array}{c} x_{d}\\ y_{d} \end{array}\right]=\left[\begin{array}{c} x+tan\left(\psi \right)\cdot y\\ tan\left(\psi \right)\cdot x+y \end{array}\right] \end{array}$$

The inner 650 × 650 pixels of each distorted image were used by cropping out the sheared edges. Each image was then filtered by a circular Hanning window, *w* (Harris, 1978).

(2) $$\begin{array}{l} w\left(r\right)=\cos^{2}\left(\frac{\pi }{N}\cdot r\right) \end{array}$$

In equation 2, r is the radial distance of the pixel position from the center of the image and *N* was set to be equal to the image dimension, i.e., 650 pixels.

Up-skewed and down-skewed adapting image sequences (Figure 1A) were prepared by skewing 12,000 natural images at a shear angle of Ψ = +25° and Ψ = −25°, respectively. During adaptation, these image sequences were rendered at a rate of 25 frames per second.

**Figure 8:**
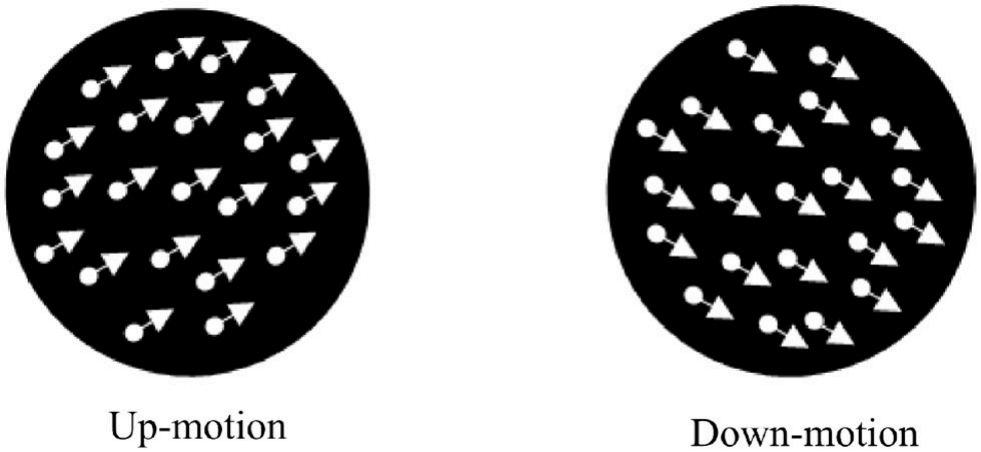
Illustration of moving random dot test stimuli.

Test stimuli were white dynamic random dots shown on a black background at a contrast of 1 in a circular annulus (Figure 8). The diameter of the annulus had the same dimension as the adapting stimuli, i.e., 650 pixels, and always consists of 2,000 dots. Each dot was circular and subtended a visual angle of 0.14°. The dots move coherently at a speed of 3°/*s*. The motion direction of the test stimuli was either diagonally up or diagonally down at an angle of θ from the horizontal (Equation 3). For a specific motion direction, θ, the position of the dots, *x*_1_ and *y*_1_ was updated to *x*_2_ and *y*_2_ in the subsequent frame using Equation (3). Positive θ corresponds to upward motion and negative to downward motion. Dots reaching the edge of the annulus were randomly repositioned to a new location within the annulus.

(3) $$\begin{array}{l} \left[\begin{array}{c} x_{2}\\ y_{2} \end{array}\right]=\left[\begin{array}{c} cos\left(\theta \right)\cdot x_{1}\\ sin\left(\theta \right)\cdot y_{1} \end{array}\right] \end{array}$$

#### Procedure

Before taking part in the psychophysical experiment, observers were informed about the procedure and trained on how to respond to the test stimuli using a keyboard. Viewing was monocular.

**Figure 9:**
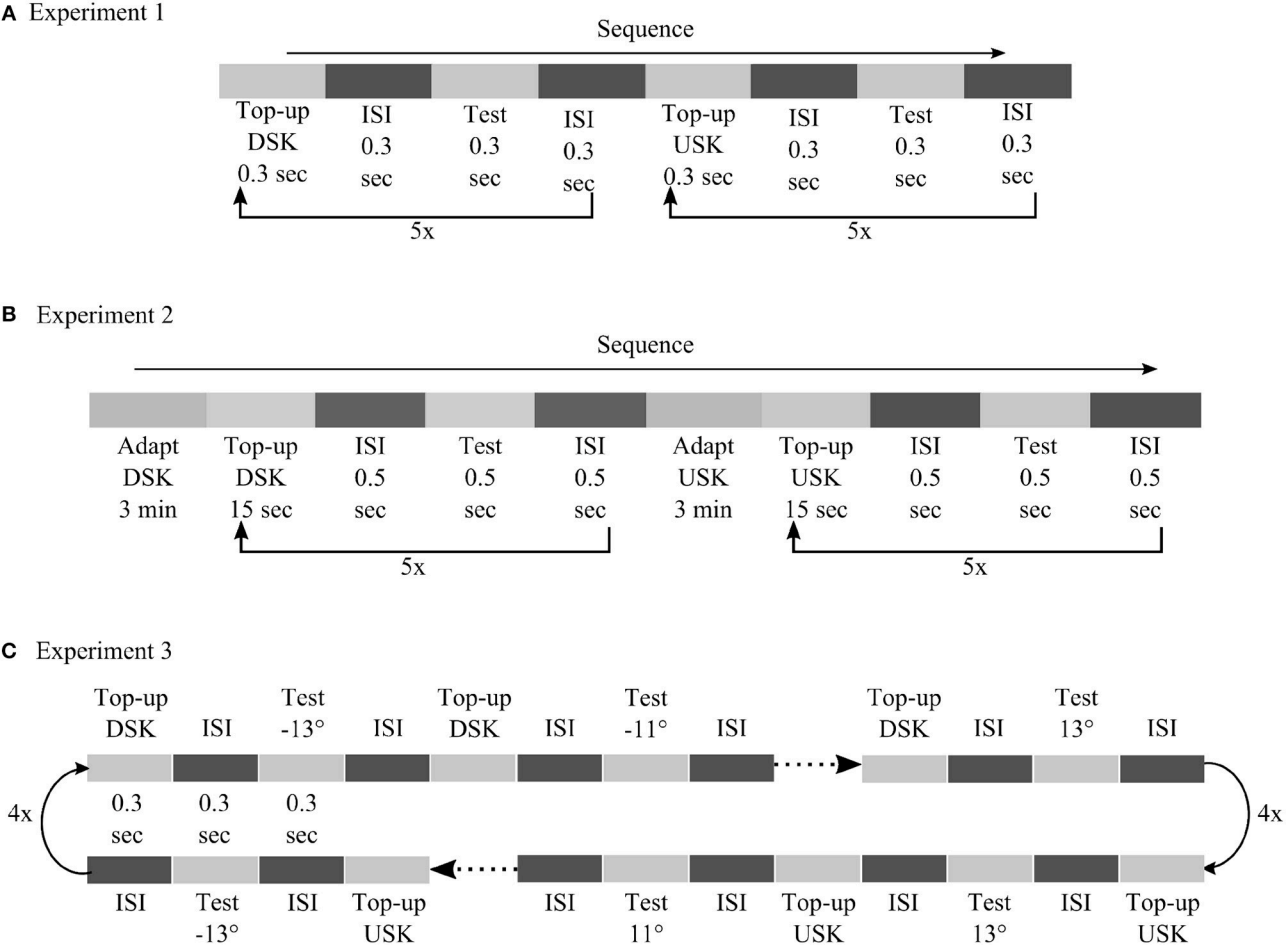
Detailed illustration of psychophysical procedures followed in experiment 1 **(A)**, experiment 2 **(B)** and experiment 3 **(C)**.

The schematic of the experimental procedure for the three psychophysical experiments is shown in Figure 9. In all the three experiments, adaptation was tested to the oppositely skewed image sequences alternately, first to the down-skewed then to the up-skewed natural image sequences. The adaptation aftereffect was tested after each adaptation using the method of constant stimuli.

In experiment 1, adaptation was tested after short exposure to skewed image sequences in a top-up procedure. Each top-up and test stimulus presentation was separated by inter stimulus interval (ISI) with blank screen, each lasting for duration of 0.3 s. In experiment 2, skewed adapting image sequence was shown first for 3 min and then for 15 s after each test stimulus presentation to top-up the adaptation. Test stimuli were presented for 0.5 s. ISI between each top-up and test stimulus presentation had 0.5 s duration. In both experiments, the motion direction of the test stimuli was in a randomized order. After each test stimulus presentation, observers had to report whether the motion direction of the random dot stimuli was diagonally up or diagonally down by pressing the up or the down key of a keyboard, respectively. In experiment 1 and experiment 2, 12 motion directions were used for the test stimuli, from −6.6° to 6.6° in step size of 1.2°. Five responses were recorded for each test stimulus motion direction. In total, 60 responses were recorded to compute the psychometric curves of each adapting skew direction.

In experiment 3, we utilized a neural hysteresis phenomena to validate our models' prediction. We tested the possible interaction between adaptation from short exposure to the skewed stimuli and hysteresis effect from sequentially changing motion direction of our test stimuli. the order of the test stimuli direction was from down (−13°) to up (13°), for the DSK adaptation, and from up (13°) to down (−13°), for the USK adaptation, in step size of 2°. Thus, any possible hysteresis effect of the test stimuli sequence is expected to induce an MAE larger than the one measured in experiment 1 with a randomized order of the test stimuli motion direction. Each top-up and test stimulus presentation was separated by ISI with blank screen, each lasting for a duration of 0.3 s. As in experiment 1 and 2, observers had to report the motion direction of the test stimuli by using the up or down key of a keyboard. Four responses were recorded per each test stimulus in four cycles. In total, 56 responses were recorded to compute the psychometric curves of DSK and USK MAEs.

#### Data Analysis

In all experiments, two psychometric curves of motion direction perception were separately computed from the responses which were recorded after exposure to up- and down- skewed natural stimuli. In each psychometric function, the percentage of upward responses as a function of motion direction of the test stimuli was fitted with a cumulative Gaussian using the Psignifit 4.0 software (asymptotes set free but assumed to be equal) (Schutt et al., 2016). The point of subjective equality (PSE), i.e., the motion direction at 50 percent of upward responses indicated the motion direction that was perceived as horizontal. The size of the magnitude of the adaptation aftereffect, Δ*PSE*, was evaluated as the difference between the PSE of the USK and DSK adaptations. The sum of the USK and DSK PSEs, ∑*PSE*, was also used to quantify any direction bias of the adaptation aftereffect from the temporal ordering of the up- and down-skew exposure. The overall aftereffect was computed by averaging the Δ*PSEs* and ∑*PSEs* from all the observers. A paired sample *t*-test was conducted to evaluate the significance of the average effects.

### Modeling

Our model focuses on motion direction tuned mechanisms in the dorsal pathway of the visual cortex, specifically V1 and MT. Motion direction processing in areas V1 and MT is modeled together with a population response readout decision layer. We suggest a dynamic short-term synaptic plasticity mechanism within FF and FB pathways between V1 and MT units to realize motion direction adaptation.

Our model extends a previously developed architecture for motion processing in areas V1 and MT (Bayerl and Neumann, 2004). Here, we outline the basic features of the model and then describe the extension made to explain visual plasticity in motion perception. The readers are referred to the previous papers for detailed description of calculations, motivation and biological plausibility of the model.

The input to the recurrent motion processing V1 and MT model units is an average motion direction signal, *R*_θ_, of either the random dot test stimuli or the adapting image sequences. For the random dot test stimulus, *R*_θ_ was a Gaussian signal centered at the corresponding test stimuli motion direction and width of 15°. For the adapting image sequences, *R*_θ_ was computed by spatiotemporal correlation technique as described in detail in the Supplementary Materials. For each image sequence, the local motion signal, *R*_*x, t*, ρ, θ_ as a function of speed and direction at each spatial location *x* and sample time *t*, is initially detected by using modified Elaborated Reichardt detectors (ERDs) (Reichardt, 1987; Bayerl and Neumann, 2004) which comprise sequential preprocessing by LGN cells, orientation selective cells and cells which compute motion energy from consecutive frames. Since the focus of this study is on direction processing and also to ease the computational load, the population response *R*_*x, t*, ρ, θ_ is simplified to *R*_*x*, θ_ by integrating over speed and time. Subsequently, direction selective V1 cells filter normalized motion direction signal, $R_{x,\theta }^{norm}$, at each location with center-surround spatial normalization. For easing the computational load in the subsequent recurrent motion direction processing stage by V1 and MT units, $R_{x,\theta }^{norm}$ is spatially integrated by inserting an average pool layer just before the recurrent stage and the input signal is simplified to only direction domain *R*_θ_. Thus, in the recurrent stage, only motion direction processing by direction selective V1 and MT cells will be considered irrespective of their spatial, temporal and speed preferences.

Model units for direction tuned cells in area V1 and MT process motion direction information in a recurrent manner via FF and FB connections. The FF connection drives the input from bottom to up cortical layers and the FB modulation enhances the lower level input matching to the higher level responses. The FF and FB excitatory connections are defined by two weighting factors; i.e., constant synaptic Gaussian weights (*G*) and dynamic synaptic efficacies (*y*). The synaptic weights can be considered as the receptive field structures and the efficacies as a probability of the presynaptic neuron to activate the post synaptic neuron depending on transmitter release.

**Figure 10:**
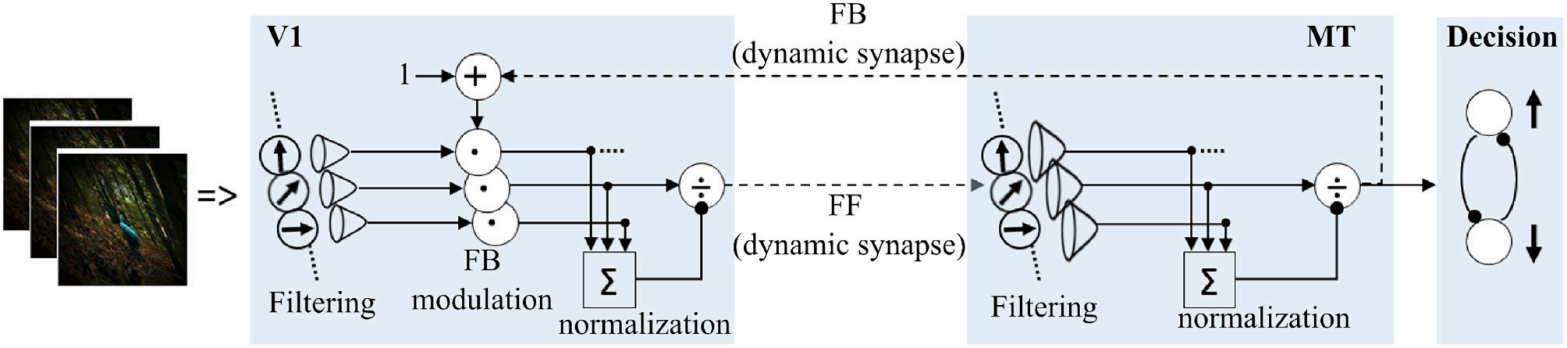
Details of motion processing in each model area. Motion is initially detected from image sequences by ERDs (Reichardt, 1987; Bayerl and Neumann, 2004), and subsequently processed in a recurrent loop in model areas V1 and MT. A steady-state model of decision-making layer is integrated to map MT responses into “upward” and “downward” motion perception decision process employed in the psychophysical experiments.

In a nutshell, as illustrated in Figure 10, motion processing in each model area constitutes three sequential stages; FF motion direction filtering, input signal enhancement with FB modulation and activity normalization with pool inhibition.

Assuming fast processes in filtering and modulating stages, the response dynamics of each model unit can be simplified to a single equation (Equation 4).

(4) $$\begin{array}{l} \tau \frac{d}{dt}v^{V1|MT}=-v^{V1|MT}+\left(1-v^{V1|MT}\right)\cdot I_{ex}^{V1|MT}-\left(1+v^{V1|MT}\right)\cdot I_{inh}^{V1|MT} \end{array}$$

(5) $$\begin{array}{l} I_{ex}^{V1}=\left(G_{c,V1}^{\theta }*R_{\theta }\right)\cdot \left(1+\lambda \cdot I_{mod}^{V1}\right) \end{array}$$

(6) $$\begin{array}{l} I_{ex}^{MT}=y^{FF}\cdot \left(G_{c,MT}^{\theta }*v^{V1}\right) \end{array}$$

(7) $$\begin{array}{l} I_{mod}^{V1}=y^{FB}\cdot v^{MT} \end{array}$$

(8) $$\begin{array}{l} I_{inh}^{V1|MT}=\frac{1}{n}\cdot \underset{\theta }{\sum }I_{ex}^{V1|MT} \end{array}$$

*y*^*FF*^ and *y*^*FB*^ are the dynamic synaptic efficacies in the FF and FB connections. *v*^*V*1^ and *v*^*MT*^ denote membrane potentials of each model unit in area V1 and MT, respectively. The membrane time constant, τ = 30 ms, was set equal in both V1 and MT model units for simplification. *I*_*ex*_, *I*_*mod*_ and *I*_*inh*_ are excitatory, modulatory and inhibitory inputs for each model unit. Since the focus of the model is only in the two stages, V1-MT, we only consider FB modulation from MT to V1. Thus, possible modulation input to area MT from higher areas, like MST and attention, would be set to zero in our model. *R*_θ_ is the driving FF inputs for model area V1 and represents the aforementioned motion direction statistics of either the adapting image sequences or the moving random dot test stimuli. $G_{c,v1}^{\theta }$ and $G_{c,MT}^{\theta }$ are the direction filtering receptive fields of V1 and MT units. For simplicity, they are assumed to be the same and implemented as a Gaussian kernel with directional width of $\sigma_{c,V1}^{\theta }=\sigma_{c,MT}^{\theta }=45^{{}^{\circ}}$, respectively. *n* = 37 represents the number of direction tuned cells in each model area and was selected randomly. Thus, with these assumptions similar input filtering and normalization processes are modeled. “*” and “·” are the convolution and scalar multiplication operators, respectively. λ = 20 is the FB strength which was adjusted to enhance the main signal over the noise, see Figure 6.

The model was extended by incorporating dynamic synaptic efficacies in the FF and FB excitatory connections to introduce synaptic adaptation in the network. Neural models of synaptic adaptation have been proposed by several researchers (Carpenter and Grossberg, 1987; Wang, 1993; Tetzlaff et al., 2012). The suggested mechanisms share common principles but also incorporate further details which depend on the particular aim of the modeling scope that is covered in addition. Here, the synaptic efficacy is described as the neurotransmitter occupancy of the presynaptic vesicle, ranging between zero and one. Previously proposed gating mechanism is considered to model the FF and FB dynamic efficacies by a first order differential equation of the form in Equations (9, 10), respectively (Carpenter and Grossberg, 1987; Hennig, 2013).

(9) $$\begin{array}{l} \tau_{syn}\cdot \frac{d}{dt}y^{FF}\left(t\right)=\alpha^{FF}\cdot \left(1-y^{FF}\left(t\right)\right)-\beta^{FF}\cdot y^{FF}\left(t\right)\cdot v^{V1}\left(t\right) \end{array}$$

(10) $$\begin{array}{l} \tau_{syn}\cdot \frac{d}{dt}y^{FB}\left(t\right)=\alpha^{FB}\cdot \left(1-y^{FB}\left(t\right)\right)-\beta^{FB}\cdot y^{FB}\left(t\right)\cdot v^{MT}\left(t\right) \end{array}$$

τ_*syn*_ = 1 is the time constant of the gating mechanism; α^*FF*^ = 1.2, β^*FF*^ = 10 are FF synaptic efficacy parameters which are fitted to replicate psychophysical results of short adaptation in experiment 1, and α^*FB*^ = 0.04, β^*FB*^ = 1.2 are FB synaptic efficacy parameters which are fitted to replicate the psychophysical results of long adaptation in experiment 2. The first term of the gating function comprises two simultaneous processes with the amount of transmitter production (α) and its inhibition −α.*y*(*t*)). Expressed differently, this term represents a tonic drive α to replenish the release pool with transmitter by an amount that is proportional to the unexcited vesicles (reserve pool), 1 − *y*(*t*). The second term denotes the transmitter release depending on the strength of presynaptic signal, *v*^*v*1^ (for the FF synapse) and *v*^*MT*^ (for the FB synapse), and the vesicle occupancy, *y*(*t*). This term depletes the presynaptic vesicle occupancy at a rate of β and regulates the strength of the temporal adaptation to form an activity-gated input. In simple terms, the synaptic strength decreases from 1 toward equilibrium state of α/(α + β) at a rate proportional to −β during continuous presynaptic excitation and restores to 1 at a rate proportional to α when the presynaptic input is off. Thus, neural responses decrease during adaptation due to the depletion of presynaptic strength and adaptation aftereffects would occur if test stimuli are presented before the depleted synaptic strength fully recovers.

#### Decision Making

In order to account for the decision process employed in the psychophysical experiments, e.g., decision-making on up or down motion direction of the test stimuli, we included a related mechanism in the model. It contains competitive steady-state responses of two output units, each representing a possible upward or downward button press motor actions. A soft-max function is used as an action selection rule to determine the probability of upward (*P*_*up*_) or downward (*P*_*down*_) output unit to win the competition between the two actions based on their values as shown in Equation (11).

(11) $$\begin{array}{l} P_{up}=\frac{exp\left(\frac{I_{up}^{ex}}{temp}\right)}{exp\left(\frac{I_{up}^{ex}}{temp}\right)+exp\left(\frac{I_{down}^{ex}}{temp}\right)}\;P_{down}=\frac{exp\left(\frac{I_{down}^{ex}}{temp}\right)}{exp\left(\frac{I_{up}^{ex}}{temp}\right)+exp\left(\frac{I_{down}^{ex}}{temp}\right)} \end{array}$$

(12) $$\begin{array}{l} I_{up}^{ex}=G_{up}^{\theta }.v^{MT}\;I_{down}^{ex}=G_{down}^{\theta }.v^{MT} \end{array}$$

$I_{up}^{ex}$ and $I_{Down}^{ex}$ are excitatory inputs to the two motor units and are weighted sum MT responses. $G_{up}^{\theta }$ and $G_{down}^{\theta }$ are Gaussian weights in the direction domain with width of 45° and their maximum centered at 90° and −90°, respectively. They can be considered as two direction channels tuned to the two directions, up and down. *temp* is a temperature parameter (Sutton and Barto, 1998) and here, it is adjusted to replicate the slope of the psychometric curves of psychophysical results. A detailed neural implementation of such decision making process is out of the scope of the paper, though it has been previously suggested (Grossberg and Pilly, 2008; Raudies and Neumann, 2010b).

#### Simulation Procedure

Identical procedures as in the real psychophysical experiments were followed in the model simulations. During adaptation, the input to the recurrent stage was the motion direction statistics, *R*_θ_, of the corresponding skewed natural image sequence. During the test phase, a direction signal centered at the corresponding test stimuli motion direction and with Gaussian noise of width 15° is fed to the recurrent motion processing stage. Figure 1 shows the input *R*_θ_ for the adapting image sequences.

From the output stage of the model, at each motion direction of the test stimulus, *P*_*up*_ corresponds to the observers' percentage of upward responses in the psychophysical measurement. *P*_*up*_ as a function of the test stimuli motion direction was then fitted with a cumulative Gaussian using Psignifit 4.0 software (asymptotes set free but assumed to be equal) (Schutt et al., 2016). The point of subjective equality (PSE), i.e., the motion direction at 50 percent of upward responses indicated the motion direction that was perceived as horizontal by the model.

#### Other Variants of the Model

Five variants of the suggested model were defined by considering specific connections and dynamic synapses using the feedback strength λ, and the synaptic constants α and β as free parameters. The FB strength λ is used to turn on or off the FB connection and the rates α and β determine the dynamics of the synaptic efficacies in the corresponding directions as follows:

- Model 1: Only FF connection with fast dynamic synapse (λ = 0, α_*FF*_ = 0.0005, β_*FF*_ = 9, *temp* = 0.00001) fitted to replicate MAE of short adaptation in experiment 1.
- Model 2: Only FF connection with slow dynamic synapse (λ = 0, α_*FF*_ = 0.5, β_*FF*_ = 10, *temp* = 0.001) fitted to replicate MAE of long adaptation in experiment 2.
- Model 3: FF-FB connection with only fast FF dynamic synapse (λ = 20, α_*FF*_ = 1, β_*FF*_ = 10, α_*FB*_ = β_*FB*_ = 0, *temp* = 0.001) fitted to replicate MAE of short adaptation in experiment 1.
- Model 4: FF-FB connection with only slow FB dynamic synapse (λ = 20, α_*FF*_ = β_*FF*_ = 0, α_*FB*_ = 0.04, β_*FB*_ = 1.2, *temp* = 0.001) fitted to replicate MAE of long adaptation in experiment 2.
- Model 5: FF-FB connection with only slow FB dynamic synapse (λ = 20, α_*FF*_ = 1, β_*FF*_ = 10, α_*FB*_ = 0.04, β_*FB*_ = 1.2, *temp* = 0.001); bringing together the fitted values of Model variant 3 and 4.

## Additional Information

KR and SW are employed by company Carl Zeiss Vision International GmbH and are scientists at the University Tübingen.

## Author Contributions

SH conducted the experiment and wrote the manuscript. SH and KR designed the experiments and analyzed the data. SH, CJ, and HN developed the model. All authors interpreted the data, contributed intellectual content to the manuscript, and approved the final submission and designed the study.

### Conflict of Interest Statement

The authors declare that the research was conducted in the absence of any commercial or financial relationships that could be construed as a potential conflict of interest.

## References

[B1] Abbott L. F. Varela J. A. Sen K. Nelson S. B. (1997). Synaptic depression and cortical gain control. Science 275, 220–224. 10.1126/science.275.5297.221 8985017

[B2] Aller T. A. (2013). Clinical management of progressive myopia. Eye 28:147. 10.1038/eye.2013.259 24357844 PMC3930271

[B3] Alvarez T. L. Kim E. H. Granger-Donetti B. (2017). Adaptation to progressive additive lenses: potential factors to consider. Sci. Rep. 7, 2529. 10.1038/s41598-017-02851-5 28566706 PMC5451391

[B4] Bao M. Engel S. A. (2012). Distinct mechanism for long-term contrast adaptation. Proc. Natl. Acad. Sci. U.S.A. 109, 5898–5903. 10.1073/pnas.1113503109 22454502 PMC3326457

[B5] Bao M. Fast E. Mesik J. Engel S. (2013). Distinct mechanisms control contrast adaptation over different timescales. J. Vis. 13, 14. 10.1167/13.10.14 23978470

[B6] Barrett J. (2004). Side Effects of Virtual Environments: A Review of the Literature. Technical report. Defense Technical Information Center.

[B7] Bashiri A. Ghazisaeedi M. Shahmoradi L. (2017). The opportunities of virtual reality in the rehabilitation of children with attention deficit hyperactivity disorder: a literature review. Korean J. Pediatr. 60, 337–343. 10.3345/kjp.2017.60.11.337 29234356 PMC5725338

[B8] Bastos A. Vezoli J. Bosman C. Schoffelen J.-M. Oostenveld R. Dowdall J.. (2015). Visual areas exert feedforward and feedback influences through distinct frequency channels. Neuron 85, 390–401. 10.1016/j.neuron.2014.12.018 25556836

[B9] Baumann T (2010). Valkaama. Available online at: http://www.valkaama.com

[B10] Bayerl P. Neumann H. (2004). Disambiguating visual motion through contextual feedback modulation. Neural Comput. 16, 2041–2066. 10.1162/0899766041732404 15333206

[B11] Bayerl P. Neumann H. (2007). Disambiguating visual motion by form-motion interaction-A computational model. Int. J. Comput. Vis. 72, 27–45. 10.1007/s11263-006-8891-8

[B12] Beck C. Neumann H. (2010). Interactions of motion and form in visual cortex-a neural model. J. Physiol. Paris 104, 61–70. 10.1016/j.jphysparis.2009.11.005 19909811

[B13] Betsch B. Y. Einhäuser W. Körding K. P. König P. (2004). The world from a cat's perspective - statistics of natural videos. Biol. Cybern. 90, 41–50. 10.1007/s00422-003-0434-6 14762723

[B14] Bex P. J. Dakin S. C. Mareschal I. (2005). Critical band masking in optic flow. Network 16, 261–284. 10.1080/09548980500289973 16411499

[B15] Bex P. J. Mareschal I. Dakin S. C. (2007). Contrast gain control in natural scenes. J. Vis. 7, 12. 10.1167/7.11.12 17997667

[B16] Bex P. J. Solomon S. G. Dakin S. C. (2009). Contrast sensitivity in natural scenes depends on edge as well as spatial frequency structure. J. Vis. 9, 1. 10.1167/9.10.1 19810782 PMC3612947

[B17] Billock V. A. de Guzman G. C. Kelso J. A. S. (2001). Fractal time and 1/f spectra in dynamic images and human vision. Physica D 148, 136–146. 10.1016/S0167-2789(00)00174-3

[B18] Blakemore C. Campbell F. W. (1969). On the existence of neurones in the human visual system selectively sensitive to the orientation and size of retinal images. J. Physiol. 203, 237–260. 10.1113/jphysiol.1969.sp008862 5821879 PMC1351526

[B19] Bouecke J. D. Tlapale E. Kornprobst P. Neumann H. (2011). Neural mechanisms of motion detection, integration, and segregation: from biology to artificial image processing systems. EURASIP J. Adv. Signal Process 2011, 1–22. 10.1155/2011/781561

[B20] Brainard D. H. (1997). The psychophysics toolbox. Spatial Vis. 10, 433–436. 10.1163/156856897X00357 9176952

[B21] Brosch T. Neumann H. (2014). Computing with a canonical neural circuits model with pool normalization and modulating feedback. Neural Comput. 26, 2735–2789. 10.1162/NECO_a_00675 25248083

[B22] Carpenter G. A. Grossberg S. (1987). Adaptation and transmitter gating in vertebrate photoreceptors. Adv. Psychol. 43, 273–310. 10.1016/S0166-4115(08)61764-1

[B23] Castellucci V. Pinsker H. Kupfermann I. Kandel E. R. (1970). Neuronal mechanisms of habituation and dishabituation of the gill-withdrawal reflex in Aplysia. Science 167, 1745–1748. 10.1126/science.167.3926.1745 5416543

[B24] Clifford C. W. Langley K. (1996). Psychophysics of motion adaptation parallels insect electrophysiology. Curr. Biol. 6, 1340–1342. 10.1016/S0960-9822(02)70721-5 8939568

[B25] Clifford C. W. G. Webster M. A. Stanley G. B. Stocker A. A. Kohn A. Sharpee T. O.. (2007). Visual adaptation: neural, psychological and computational aspects. Vis. Res. 47, 3125–3131. 10.1016/j.visres.2007.08.023 17936871

[B26] Clifford C. W. G. Wenderoth P. Spehar B. (2000). A functional angle on some after-effects in cortical vision. Proc. R. Soc. Lond. B Biol. Sci. 267, 1705–1710. 10.1098/rspb.2000.1198 12233765 PMC1690741

[B27] David S. V. Vinje W. E. Gallant J. L. (2004). Natural stimulus statistics alter the receptive field structure of v1 neurons. J. Neurosci. 24, 6991–7006. 10.1523/JNEUROSCI.1422-04.2004 15295035 PMC6729594

[B28] Destexhe A. Mainen Z. F. Sejnowski T. J. (1998). Kinetic models of synaptic transmission. Methods Neuronal Model. 2, 1–25.

[B29] Dong D. W. Atick J. J. (1995). Statistics of natural time-varying images. Network 6, 345–358. 10.1088/0954-898X_6_3_003

[B30] Eckert M. P. Zeil J. (2001). Towards an ecology of motion vision, in Motion Vision, eds Zanker J. M. Zeil J. (Berlin; Heidelberg: Springer), 333–369.

[B31] Edwards M. Crane M. F. (2007). Motion streaks improve motion detection. Vis. Res. 47, 828–833. 10.1016/j.visres.2006.12.005 17258262

[B32] Fang F. Murray S. O. Kersten D. He S. (2005). Orientation-tuned fMRI adaptation in human visual cortex. J. Neurophysiol. 94, 4188–4195. 10.1152/jn.00378.2005 16120668

[B33] Felleman D. J. Van D. C. E. (1991). Distributed hierarchical processing in the primate cerebral cortex. Cereb. Cortex 1, 1–47. 10.1093/cercor/1.1.1 1822724

[B34] Felsen G. Dan Y. (2005). A natural approach to studying vision. Nat. Neurosci. 8, 1643–1646. 10.1038/nn1608 16306891

[B35] Friston K. J. Büchel C. (2000). Attentional modulation of effective connectivity from V2 to V5/MT in humans. Proc. Natl. Acad. Sci. U.S.A. 97, 7591–7596. 10.1073/pnas.97.13.7591 10861020 PMC16590

[B36] Geisler W. S. (1999). Motion streaks provide a spatial code for motion direction. Nature 400, 65–69. 10.1038/21886 10403249

[B37] Geisler W. S. (2008). Visual perception and the statistical properties of natural scenes. Ann. Rev. Psychol. 59, 167–192. 10.1146/annurev.psych.58.110405.085632 17705683

[B38] Grossberg S.(ed.). (1982). How does a brain build a cognitive code?, in Studies of Mind and Brain (Dordrecht: Springer), 1–52.

[B39] Grossberg S. Pilly P. K. (2008). Temporal dynamics of decision-making during motion perception in the visual cortex. Vis. Res. 48, 1345–1373. 10.1016/j.visres.2008.02.019 18452967

[B40] Grzywacz N. M. de Juan J. (2003). Sensory adaptation as Kalman filtering: theory and illustration with contrast adaptation. Network 14, 465–482. 10.1088/0954-898X_14_3_305 12938767

[B41] Habtegiorgis S. W. Rifai K. Lappe M. Wahl S. (2017). Adaptation to Skew Distortions of Natural Scenes and Retinal Specificity of Its Aftereffects. Front. Psychol. 8:1158. 10.3389/fpsyg.2017.01158 28751870 PMC5508008

[B42] Harris F. J. (1978). On the use of windows for harmonic analysis with the discrete Fourier transform. Proc. IEEE 66, 51–83. 10.1109/PROC.1978.10837

[B43] Hawkins R. D. Kandel E. R. Siegelbaum S. A. (1993). Learning to modulate transmitter release: themes and variations in synaptic plasticity. Ann. Rev. Neurosci. 16, 625–665. 10.1146/annurev.ne.16.030193.003205 8096376

[B44] Hennig M. H. (2013). Theoretical models of synaptic short term plasticity. Front. Comput. Neurosci. 7:154 10.3389/fncom.2013.00154 24198783 PMC3812535

[B45] Holden BA Fricke TR Ho SM Wong R Schlenther G Cronjé S. (2008). Global vision impairment due to uncorrected presbyopia. Arch. Ophthalmol. 126, 1731–1739. 10.1001/archopht.126.12.1731 19064856

[B46] Hupe J.-M. James A. C. Girard P. Lomber S. G. Payne B. R. Bullier J. (2001). Feedback connections act on the early part of the responses in monkey visual cortex. J. Neurophysiol. 85, 134–145. 10.1152/jn.2001.85.1.134 11152714

[B47] Jääskeläinen I. P. Ahveninen J. Andermann M. L. Belliveau J. W. Raij T. Sams M. (2011). Short-term plasticity as a neural mechanism supporting memory and attentional functions. Brain Res. 1422, 66–81. 10.1016/j.brainres.2011.09.031 21985958 PMC3220931

[B48] Johnson L. Buckley J. G. Scally A. J. Elliott D. B. (2007). Multifocal spectacles increase variability in toe clearance and risk of tripping in the elderly. Invest. Ophthalmol. Vis. Sci. 48, 1466–1471. 10.1167/iovs.06-0586 17389472

[B49] Kayser C. Kording K. P. Konig P. (2004). Processing of complex stimuli and natural scenes in the visual cortex. Curr. Opin. Neurobiol. 14, 468–473. 10.1016/j.conb.2004.06.002 15302353

[B50] Keshner E. A. (2004). Virtual reality and physical rehabilitation: a new toy or a new research and rehabilitation tool? J. Neuroeng. Rehabil. 1:8 10.1186/1743-0003-1-8 15679943 PMC546404

[B51] Kohn A. (2007). Visual adaptation: physiology, mechanisms, and functional benefits. J. Neurophysiol. 97, 3155–3164. 10.1152/jn.00086.2007 17344377

[B52] Kording K. P. Tenenbaum J. B. Shadmehr R. (2007). The dynamics of memory as a consequence of optimal adaptation to a changing body. Nat. Neurosci. 10, 779–786. 10.1038/nn1901 17496891 PMC2551734

[B53] Laver K. George S. Thomas S. Deutsch J. E. Crotty M. (2012). Virtual reality for stroke rehabilitation. Stroke 43, e20–e21. 10.1161/STROKEAHA.111.642439 22713539

[B54] MacLeod K. Ashida G. Glaze C. Carr C. (2010). Short-term synaptic plasticity and adaptation contribute to the coding of timing and intensity information, in The Neurophysiological Bases of Auditory Perception, eds Lopez-Poveda E. A. Palmer A. R. Meddis R. (New York, NY: Springer), 347–356.

[B55] Markram H. Toledo-Rodriguez M. Wang Y. Gupta A. Silberberg G. Wu C. (2004). Interneurons of the neocortical inhibitory system. Nat. Rev. Neurosci. 5, 793. 10.1038/nrn1519 15378039

[B56] Mather G. Pavan A. Campana G. Casco C. (2008). The motion aftereffect reloaded. Trends Cogn. Sci. 12, 481–487. 10.1016/j.tics.2008.09.002 18951829 PMC3087115

[B57] Mather G. Verstraten F. Anstis S. M. (1998). The Motion Aftereffect: A Modern Perspective. Cambridge, MA: MIT Press. 10.1016/s1364-6613(98)01142-5 21227087

[B58] Mei G. Dong X. Dong B. Bao M. (2015). Spontaneous recovery of effects of contrast adaptation without awareness. Front. Psychol. 6:1464. 10.3389/fpsyg.2015.01464 26483723 PMC4588121

[B59] Meister D. J. Fisher S. W. (2008). Progress in the spectacle correction of presbyopia. Part 1: design and development of progressive lenses. Clin. Exp. Optom. 91, 240–250. 10.1111/j.1444-0938.2007.00245.x 18279415

[B60] Mesik J. Bao M. Engel S. A. (2013). Spontaneous recovery of motion and face aftereffects. Vis. Res. 89, 72–78. 10.1016/j.visres.2013.07.004 23872167

[B61] Monier C. Fournier J. Frégnac Y. (2008). *In vitro* and *in vivo* measures of evoked excitatory and inhibitory conductance dynamics in sensory cortices. J. Neurosci. Methods 169, 323–365. 10.1016/j.jneumeth.2007.11.008 18215425

[B62] Pack C. C. Livingstone M. S. Duffy K. R. Born R. T. (2003). End-stopping and the aperture problem: two-dimensional motion signals in macaque V1. Neuron 39, 671–680. 10.1016/S0896-6273(03)00439-2 12925280

[B63] Patterson C. A. Duijnhouwer J. Wissig S. C. Krekelberg B. Kohn A. (2013). Similar adaptation effects in primary visual cortex and area MT of the macaque monkey under matched stimulus conditions. J. Neurophysiol. 111, 1203–1213. 10.1152/jn.00030.2013 24371295 PMC3949312

[B64] Pavan A. Marotti R. B. Mather G. (2013). Motion-form interactions beyond the motion integration level: evidence for interactions between orientation and optic flow signals. J. Vis. 13, 16. 10.1167/13.6.16 23729767 PMC3670578

[B65] Raudies F. Neumann H. (2010a). A model of neural mechanisms in monocular transparent motion perception. J. Physiol. Paris 104, 71–83. 10.1016/j.jphysparis.2009.11.010 19900543

[B66] Raudies F. Neumann H. (2010b). A neural model of the temporal dynamics of figure–ground segregation in motion perception. Neural Netw. 23, 160–176. 10.1016/j.neunet.2009.10.005 19931405

[B67] Regehr W. G. (2012). Short-term presynaptic plasticity. Cold Spring Harb. Perspect. Biol. 4:a005702. 10.1101/cshperspect.a005702 22751149 PMC3385958

[B68] Reichardt W. (1987). Evaluation of optical motion information by movement detectors. J. Comp. Physiol. A 161, 533–547. 10.1007/BF00603660 3681769

[B69] Ringach D. L. Hawken M. J. Shapley R. (2002). Receptive field structure of neurons in monkey primary visual cortex revealed by stimulation with natural image sequences. J. Vis. 2, 2. 10.1167/2.1.2 12678594

[B70] Rokszin A. Márkus Z. Braunitzer G. Berényi A. Benedek G. Nagy A. (2010). Visual pathways serving motion detection in the mammalian brain. Sensors 10, 3218–3242. 10.3390/s100403218 22319295 PMC3274219

[B71] Roth A. van Rossum M. C. (2009). Modeling synapses. Comput. Model. Methods Neurosci. 6, 139–160. 10.7551/mitpress/9780262013277.003.0007

[B72] Schutt H. H. Harmeling S. Macke J. H. Wichmann F. A. (2016). Painfree and accurate Bayesian estimation of psychometric functions for (potentially) overdispersed data. Vis. Res. 122, 105–123. 10.1016/j.visres.2016.02.002 27013261

[B73] Sheedy J. E. Andre B. (2005). Chapter 44: Prescribing multifocal lenses in Duane's *Clinical Ophthalmology*, eds Tasman W. Jaeger E. A. (Philadelphia, PA: Lippincott-Raven), 6–12.

[B74] Sillito A. M. Cudeiro J. Jones H. E. (2006). Always returning: feedback and sensory processing in visual cortex and thalamus. Trends Neurosci. 29, 307–316. 10.1016/j.tins.2006.05.001 16713635

[B75] Simoncelli E. P. (2003). Vision and the statistics of the visual environment. Curr. Opin. Neurobiol. 13, 144–149. 10.1016/S0959-4388(03)00047-3 12744966

[B76] Snow M. Coen-Cagli R. Schwartz O. (2017). Adaptation in the visual cortex: a case for probing neuronal populations with natural stimuli. F1000Research 6:1246. 10.12688/f1000research.11154.1 29034079 PMC5532795

[B77] Stevenson I. H. Cronin B. Sur M. Kording K. P. (2010). Sensory adaptation and short term plasticity as bayesian correction for a changing brain. PLoS ONE 5:e12436. 10.1371/journal.pone.0012436 20865056 PMC2928744

[B78] Stuit S. M. (2009). Motion Processing, Adaptation and Aftereffects: A Review. Ph.D. thesis, Utrecht University.

[B79] Sutton R. S. Barto A. G. (1998). Reinforcement Learning: An Introduction, Vol. 1. Cambridge, MA: MIT Press Cambridge.

[B80] Tetzlaff C. Kolodziejski C. Markelic I. Wörgötter F. (2012). Time scales of memory, learning, and plasticity. Biol. Cybern. 106, 715–726. 10.1007/s00422-012-0529-z 23160712

[B81] Thiele A. (2012). Nmda receptors figure it out. Proc. Natl. Acad. Sci. U.S.A. 109, 10749–10750. 10.1073/pnas.1207975109 22733739 PMC3390867

[B82] Tsodyks M. Pawelzik K. Markram H. (1998). Neural networks with dynamic synapses. Neural Comput. 10, 821–835. 10.1162/089976698300017502 9573407

[B83] Ullman S. (1995). Sequence seeking and counter streams: a computational model for bidirectional information flow in the visual cortex. Cereb. Cortex 5, 1–11. 10.1093/cercor/5.1.1 7719126

[B84] van de Grind W. A. Lankheet M. J. Tao R. (2003). A gain-control model relating nulling results to the duration of dynamic motion aftereffects. Vis. Res. 43, 117–133. 10.1016/S0042-6989(02)00495-9 12536135

[B85] van Rossum M. C. van der Meer M. A. Xiao D. Oram M. W. (2008). Adaptive integration in the visual cortex by depressing recurrent cortical circuits. Neural Comput. 20, 1847–1872. 10.1162/neco.2008.06-07-546 18336081

[B86] Wang D. (1993). A neural model of synaptic plasticity underlying short-term and long-term habituation. Adapt. Behav. 2, 111–129. 10.1177/105971239300200201

[B87] Wark B. Fairhall A. Rieke F. (2009). Timescales of inference in visual adaptation. Neuron 61, 750–761. 10.1016/j.neuron.2009.01.019 19285471 PMC2677143

[B88] Wark B. Lundstrom B. N. Fairhall A. (2007). Sensory adaptation. Curr. Opin. Neurobiol. 17, 423–429. 10.1016/j.conb.2007.07.001 17714934 PMC2084204

[B89] Webster M. A. (2011). Adaptation and visual coding. J. Vis. 11, 3. 10.1167/11.5.3 21602298 PMC3245980

[B90] Webster M. A. (2015). Visual adaptation. Ann. Rev. Vis. Sci. 1, 547–567. 10.1146/annurev-vision-082114-035509 26858985 PMC4742349

[B91] Williams D. Phillips G. (1987). Cooperative phenomena in the perception of motion direction. JOSA A 4, 878–885. 10.1364/JOSAA.4.000878 3598741

[B92] Yao R. Heath T. Davies A. Forsyth T. Mitchell N. Hoberman P. (2014). Oculus vr Best Practices Guide. Menlo Park, CA: Oculus VR 27–39.

[B93] York L. C. Van Rossum M. C. (2009). Recurrent networks with short term synaptic depression. J. Comput. Neurosci. 27, 607. 10.1007/s10827-009-0172-4 19578989

[B94] Zucker R. S. Regehr W. G. (2002). Short-term synaptic plasticity. Ann. Rev. Physiol. 64, 355–405. 10.1146/annurev.physiol.64.092501.114547 11826273

