# Supplementary material for: The Role of Bottom-Up and Top-Down Cortical Interactions in Adaptation to Natural Scene Statistics
Source: Front Neural Circuits. 2019 Feb 13;13:9. doi: 10.3389/fncir.2019.00009 (PMC6381060; doi:10.3389/fncir.2019.00009)

Supplementary material

1. Motion detection from natural image sequences

At each location, local motion direction was measured from input image sequences with correlation-based motion detection using modified elaborated Reichardt detectors (ERDs) [35, 36]. This input stage consists of three steps.

1. The first is center surround Gaussian filtering mimicking contrast detection by LGN cells.

$$\boldsymbol{r^{lgn}(x,t)} = \boldsymbol{I(x,t)} * (\boldsymbol{G_c(x)} - \boldsymbol{G_s(x)}) \quad \textbf{(S1)}$$

r^{lgn} is LGN response at each location x and time t. $G_c \; and \; G_s$ are the center and surround isotropic Gaussian filters with width of 0.1° and 0.3° in space domain, respectively.

2. The second step is static oriented contrast detection by orientation selective complex cells at a fixed frequency and independent of polarity. This is realized by oriented Gabor filter responses with shunting normalization.

$$\boldsymbol{r'_{x,t,\phi}} = \left(\boldsymbol{r^{lgn}} * \boldsymbol{G_{x_{odd}(\phi)}}\right)^2 + \left(\boldsymbol{r^{lgn}} * \boldsymbol{G_{x_{even}(\phi)}}\right)^2 \quad \textbf{(S2)}$$

$$r_{x,t,\phi} = \frac{r'_{x,t,\phi}}{0.01 + \left(\sum_\phi r'_{x,t,\phi} * G_{surr}(x,y)\right)^2} \quad \textbf{(S3)}$$

$G_{x_{odd}(\phi)}$ and $G_{x_{even}(\phi)}$ are sine and cosine Gabor filters, respectively, with isotropic width of 1°, spatial frequency of 1.57 cpd and oriented at ϕ. $r_{x,t,\phi}$ is the filter response normalized by the surround field G_s of width 5° at each location and time. Oriented contrasts were computed in eight orientations; ϕ = 0, 22.5°...180°.

3. The third step consists of responses of direction selective cells which compute orientation-independent motion energy between two consecutive frames. These mechanisms pool over responses of all orientation selective cells at different time steps.

$$\boldsymbol{r^{+}_{x,t,\rho,\theta}} = \sum_{\phi} \left(\boldsymbol{r_{x,t,\phi}}.\boldsymbol{r_{x+\Delta x,t+\Delta t,\phi}}\right) * \boldsymbol{G_c} \quad \textbf{(S4)}$$

$$r^{-}_{x,t,\rho,\theta} = \sum_{\phi} \left(r_{x+\Delta x,t,\phi}.r_{x,t+\Delta t,\phi}\right) * G_c \quad \textbf{(S5)}$$

$$R_{x,t,\rho,\theta} = \frac{r^{+}_{x,t,\theta} - 0.5.r^{-}_{x,t,\rho,\theta}}{1 + \lfloor r^{-}_{x,t,\rho,\theta} \rfloor_{+}} \quad \textbf{(S6)}$$

r^{+} and r^{-} are orientation independent responses of half-detectors for a specific velocity defined by a shift $\Delta x = (\rho(speed), \theta(direction))$ between consecutive frames and pooled over small spatial neighborhood by isotropic Gaussian field (G_c) of width 1°. Thus, they can be considered as raw correlation of outputs of complex cells. $R_{x,t,\theta}$ indicates the population response normalized by the opponent signal at each location and time. $\max(.,0)$ non-linear operation is realized by a half wave rectification operator $\lfloor . \rfloor_{+}$.

We computed $R_{x,t,\rho,\theta}$ for each natural image sequences used in the psychophysical experiments in five speed ranges, $\rho = 1,2,3,4,5 \; dps$, and 36 direction ranges, $\theta = -180°, 170° \dots 180°$. Since the

focus of the study is only motion direction, the population response was integrated over time and speed domain as in equation (S7).

$$R_{x,\theta} = \sum_{t} \sum_{\rho} R_{x,t,\rho,\theta} \tag{S7}$$

Subsequently, at each location, the signal is detected by direction selective V1 cells with center-surround shunting normalization as follows.

$$R_{x,\theta}^{norm} = \frac{R_{x,\theta} * G_c^x}{0.01 + \left(\sum_\theta R_{x,\theta} * G_{surr}^x\right)^2} \tag{S8}$$

$R_{x,\theta}^{norm}$ is the normalized direction signal at each location. G_c^x and G_s^x are the center and surround fields with Gaussian width of 1° and 5°, respectively.

For computational load purpose, we averaged the $R_{x,\theta}^{norm}$ in space domain by inserting an average pool layer just before the recurrent stage as in equation (S9). Thus, the averaged population response, R_θ, for each adapting skewed natural image sequence represents the average motion direction statistics.

$$R_\theta = \frac{\sum_x R_{x,\theta}^{norm}}{n_x} \tag{S9}$$

Where $n_x = 650 \times 650$ is the number of pixels in each image frame.

For all the simulations in this paper, R_θ is used as an input to the higher level model areas during exposure to skewed natural image sequences.

2. Psychometric curves of model predictions

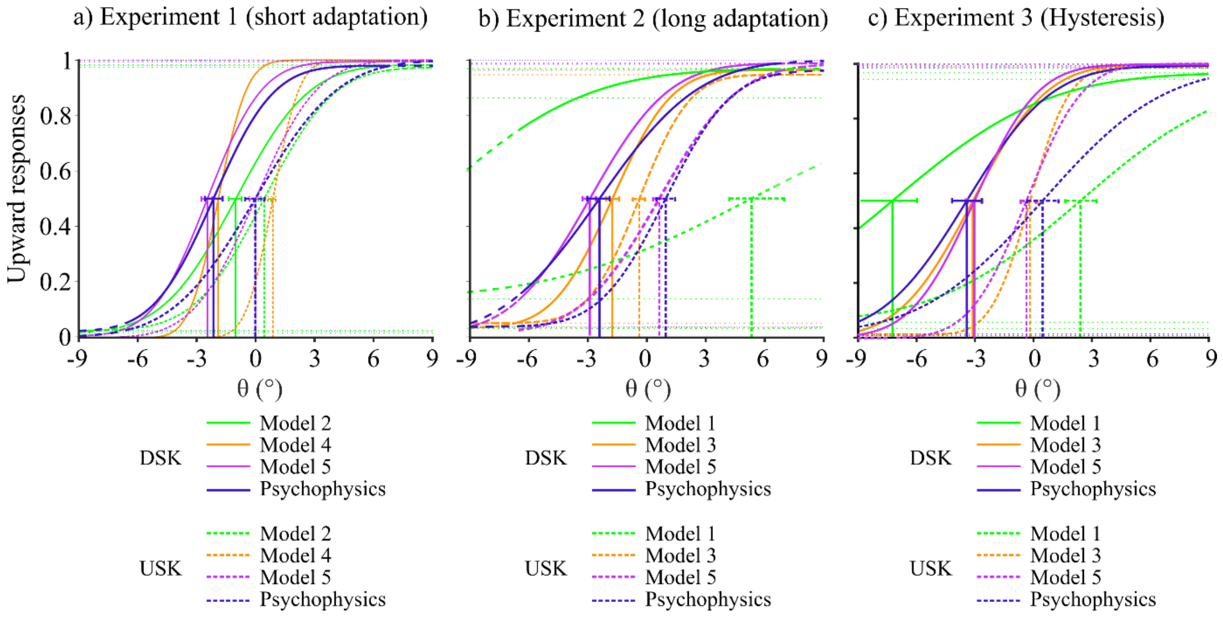

Supplement: Supplementary file 1 [file Data_Sheet_1.pdf]
